# *In Vitro* and *In Vivo* Studies for Assessing the Immune Response and Protection-Inducing Ability Conferred by *Fasciola hepatica*-Derived Synthetic Peptides Containing B- and T-Cell Epitopes

**DOI:** 10.1371/journal.pone.0105323

**Published:** 2014-08-14

**Authors:** Jose Rojas-Caraballo, Julio López-Abán, Luis Pérez del Villar, Carolina Vizcaíno, Belén Vicente, Pedro Fernández-Soto, Esther del Olmo, Manuel Alfonso Patarroyo, Antonio Muro

**Affiliations:** 1 Parasite and Molecular Immunology Laboratory, Tropical Disease Research Centre, Universidad de Salamanca (IBSAL-CIETUS), Salamanca, Spain; 2 Molecular Biology and Immunology Department, Fundación Instituto de Inmunología de Colombia (FIDIC), Bogotá, Colombia; 3 Pharmaceutical Chemistry Department, Tropical Disease Research Centre, Universidad de Salamanca (IBSAL-CIETUS), Salamanca, Spain; 4 Basic Sciences Department, School of Medicine and Health Sciences, Universidad del Rosario, Bogotá, Colombia; Centro de Investigacion y de Estudios Avanzados del Instituto Politecnico Nacional, Mexico

## Abstract

Fasciolosis is considered the most widespread trematode disease affecting grazing animals around the world; it is currently recognised by the World Health Organisation as an emergent human pathogen. Triclabendazole is still the most effective drug against this disease; however, resistant strains have appeared and developing an effective vaccine against this disease has increasingly become a priority. Several bioinformatics tools were here used for predicting B- and T-cell epitopes according to the available data for *Fasciola hepatica* protein amino acid sequences. BALB/c mice were immunised with the synthetic peptides by using the ADAD vaccination system and several immune response parameters were measured (antibody titres, cytokine levels, T-cell populations) to evaluate their ability to elicit an immune response. Based on the immunogenicity results so obtained, seven peptides were selected to assess their protection-inducing ability against experimental infection with *F. hepatica* metacercariae. Twenty-four B- or T-epitope-containing peptides were predicted and chemically synthesised. Immunisation of mice with peptides so-called B1, B2, B5, B6, T14, T15 and T16 induced high levels of total IgG, IgG1 and IgG2a (p<0.05) and a mixed Th1/Th2/Th17/Treg immune response, according to IFN-γ, IL-4, IL-17 and IL-10 levels, accompanied by increased CD62L^+^ T-cell populations. A high level of protection was obtained in mice vaccinated with peptides B2, B5, B6 and T15 formulated in the ADAD vaccination system with the AA0029 immunomodulator. The bioinformatics approach used in the present study led to the identification of seven peptides as vaccine candidates against the infection caused by *Fasciola hepatica* (a liver-fluke trematode). However, vaccine efficacy must be evaluated in other host species, including those having veterinary importance.

## Introduction

Fasciolosis is one of the most important helminthiasis worldwide affecting grazing livestock due its widespread geographical distribution and resulting economic loss; it is caused by the common liver fluke *Fasciola hepatica,* along with the related species *Fasciola gigantica* [1]. Besides being a well-known veterinary problem, fasciolosis has also recently become considered as an emerging parasitic human disease, having a significant impact on public health, causing millions of people to be at risk of infection. Reports have indicated its increase in many Latin-American, African, European and Asian countries [2], [3]. Taking its impact on human health and wide emergence into account, human fasciolosis has been recently included in the World Health Organization’s (WHO) list of priorities related to Neglected Tropical Diseases [4].

It is well-known that methodological and technical difficulties related to diagnosis have limited progress in combating human fasciolosis globally, including drawbacks in diagnosing infection and assessing drug efficacy and resistance, mainly concerning triclabendazole which is still the most effective drug for combating the disease. Indeed, no commercial vaccine is currently available and developing vaccines for controlling animal and human fasciolosis thus represents a tremendous research opportunity. Many candidate proteins have been tested for a long time now as target antigens in vaccination assays against fluke, including fatty acid-binding proteins, glutathione S-transferases, cathepsin proteases, leucine aminopeptidase, fluke haemoglobin and thioredoxin peroxidase. However, no consensus regarding the factors required for immunological protection has yet emerged and there has been no report to date of a successful field trial concerning a liver fluke vaccine [5], [6]. Many factors may be responsible for the failure of these vaccines when tested; vaccine formulation, choice of adjuvant and delivery route and dosage will affect the way in which different animals and breeds will respond to different vaccines and, possibly, the choice of target antigen. The aforementioned research challenge must thus involve identifying new target antigens for obtaining an effective vaccine against *F. hepatica* [7].

Public access to an increasing number of pathogen genomes which have been totally or partially sequenced, along with the use of powerful *in silico* analysis, currently relies on rapidly screening a large number of expressed pathogen proteins for their ability to induce a protective immune response; vaccine candidates based on genome information has thus become possible [8]. Synthetic peptide-based vaccines, in which small peptides derived from known target epitopes are used to induce an immune reaction, have thus attracted interest as a promising approach to treating several infectious diseases and tumours, since they have several advantages over other forms of vaccine, particularly regarding safety, ease of production, reproducibility, low cost and ensuring a more effective antigen-specific immune response to a particular cell type [9].

As epitope-based vaccines only contain small sequences derived from an entire protein known to bind to various major histocompatibility complex (MHC) molecules, predicting peptide-MHC binding and mapping epitopes are crucial in their design [10], [11]. This approach has led to identifying specific binding motifs for effectively predicting both B- and T-cell epitopes. There are several online-based tools for predicting the MHC-peptide interaction available for researchers, although B-cell epitope mapping algorithms have lagged behind T-cell ones and only a few B-cell epitope mapping algorithms are in current use [11]; this is because there are still several obstacles to developing B-cell epitope prediction for peptide-based vaccine design [12].

Synthetic peptides have been examined as potential prophylactic vaccines against viral, bacterial and parasitic diseases for many years now [13], [14] and as therapeutic vaccines for chronic infections and non-infectious diseases, as well as cancer [15]. Despite such a large number of potential synthetic peptides having been identified, none are currently being marketed for human use [16] and few studies reported to date have used synthetic peptides as anti-helminth vaccines, including *Echinococcus granulosus* [17], *Trichinella spiralis* [18], *Brugia malayi* [19], *Taenia solium* [20], *Schistosoma mansoni* [21] and *F. gigantica* [22]. Regarding *F. hepatica*, synthetic peptides have been used in diagnosing human infections. In the search for selecting optimal vaccine candidate proteins expressed by *F. hepatica* and trigger an immune response induced by previously reported candidate proteins, our group has focused on the rational identification of B- and T-cell epitopes by *in silico* mapping.

Several peptides have thus been chemically-synthesised and then assessed using *in vitro* and *in vivo* assays to evaluate the induced immune response and their inducing-protection ability. Our trials have involved using a murine model prepared with an adjuvant/adaptation (ADAD) vaccination system [23] and then immunised with a chosen peptide antigen, a natural immunomodulator extracted from the rhizome of the fern *Phlebodium pseudoaureum* (PAL) or a chemically-synthesised aliphatic diamine immunomodulator AA0029 [24] and a non-haemolytic adjuvant containing *Quillaja saponaria* (QS) saponins to form an emulsion with a non-mineral oil in a 70/30 oil/water ratio. This study was aimed at selecting peptides containing B- and T-cell epitopes, assessing their immunogenicity, and testing the protection-inducing ability against experimental infection with *F. hepatica* metacercariae of the highly immunogenic ones.

## Materials and Methods

### Ethics statement and experimental animals

The animal procedures in this study complied with Spanish (Real Decreto RD53/2013) and European Union (European Directive 2010/63/EU) guidelines regarding animal experimentation for the protection and humane use of laboratory animals, and were conducted at the University of Salamanca’s accredited Animal Experimentation Facilities (Register number: PAE/SA/001). University of Salamanca’s Ethics Committee approved procedures used in the present study (protocol approval number 48531). Seven-week-old female BALB/c and CD1 mice (Charles River Laboratories, Barcelona, Spain) weighing 20 to 22 g were used for the experiments. Animals were kept in plastic boxes with food and water *ad libitum* at the University of Salamanca’s Animal Experimentation Facilities. Animal care involved regular 12 h light–dark periods at 20°C. All efforts to minimise suffering were made.

### Fasciola hepatica sequence selection

Due to a lack of data regarding the *F. hepatica* complete genome sequence, only partial information concerning several genes and protein sequences was available in pertinent databases at the beginning of the present study. Each sequence obtained was individually predicted for signal peptide (SP) and trans-membrane (TM) domains to select secreted proteins. SP was predicted with the SignalP 3.0 server [25] available at (http://www.cbs.dtu.dk/services/SignalP/) and the TM domain was predicted using the TMHMM v.2.0 server (http://www.cbs.dtu.dk/services/TMHMM/). Only proteins showing a SP and no TM domains were finally selected and grouped into families. All selected sequences were subjected to multiple sequence alignment using ClustalW (http://npsa-pbil.ibcp.fr) and conserved or semi-conserved fragments were chosen for B- and T-cell epitope prediction.

### B- and T-cell epitope prediction

The BepiPred method was used for predicting linear B-cell epitopes [26]; the server (BepiPred 1.0) and training datasets are publicly available at http://www.cbs.dtu.dk/services/BepiPred. This method involves each amino acid receiving a prediction score based on Hidden Markov Model (HMM) profiles for known antigens and incorporates propensity scale methods based on hydrophilicity and secondary structure prediction. Predicted linear B-cell epitopes were then compared to the values given for each amino acid using ANTHEPROT 3D software (http://antheprot-pbil.ibcp.fr) regarding several physical-chemical profiles, such us antigenicity, hydrophobicity, flexibility and solvent accessibility [27]. The values obtained for each profile were averaged in groups of 20 amino acids. Only regions showing the best probability for each protein (score ≥0.5, according to the HMM result) were selected as promising linear B-cell epitopes.

The SYFPEITHI database [28], freely accessible at http://www.syfpeithi.de/was used as the source of MHC class II binding peptides. SYFPEITHI allows predicting peptide binding to a defined MHC type and predictions were made for H2-Ad murine MHC class-II ligands. The analysis was performed choosing 15-mer (15 amino acids) for MHC type II as prediction parameter. The resulting peptides showing the highest scores in the dataset capable of binding to the murine class-II molecules described above were selected as candidate epitopes.

### Peptide synthesis

All derived peptides selected on the basis of B- or T-cell epitope predictions for each protein were chemically synthesised (Fundación Instituto de Inmunología, FIDIC, Colombia) by the solid-phase peptide synthesis according to the methodology first described by Merrifield [29] and subsequently modified by Houghten [30] using the t-Boc strategy and a BHA (benzyhydrylamine) resin (0.7 meq/mg). One cysteine and one glycine residue were added at both amino and carboxyl-terminal extremes to allow their polymerisation via oxidisation. Peptides were purified by reverse phase high performance liquid chromatography (to at least >90% purity), characterised by MALDI-TOF mass spectrometry, lyophilised and quantified. Freeze-dried synthesised peptides were used in the ensuing experiments.

### Cytotoxicity evaluation

The J774.2 mouse peritoneal macrophage cell line was used in this study; it was grown in RPMI-1640 culture medium supplemented with 10% FBS, 2 mM L-glutamine, 100 U/mL penicillin, and 100 µg/mL streptomycin, at 37°C in humidified 95% air and 5% CO_2_. J774.2 peritoneal macrophage cells were plated in complete RPMI-1640 culture medium at 1×10^6^ cells/well concentration in 12-well culture plates (Costar, Cambridge, MA), and left to adhere for 2 h at 37°C in 5% CO_2_. Non-adhering cells were removed by gentle washing with complete RPMI-1640 culture medium. Adherent J774.2 cells were incubated with each synthetic peptide at different concentrations (1–100 µg/mL). After 48 h incubation at 37°C in 5% CO_2_, supernatants were removed and cell viability was measured on adhered cells by MTT (3-(4,5-dimethylthiazole-2-yl)-2,5-diphenyltetrazolium bromide) assay, measuring the absorbance at 540 nm. Controls for checking solvent cytotoxicity were also included.

### Vaccine formulation and immunisation trial

One hundred and sixty-eight female BALB/c mice (56 groups of 3 mice per group) were used in this study. The immune response in mice was studied in two separate experiments (A and B). Experiment A: group 1 (untreated control group; n = 6), group 2 (ADAD and natural immunomodulator PAL [ADADn]; n = 6), groups 3–14 (ADADn together with B1–B12 B-epitope-containing peptides; n = 36) and groups 15–26 (ADADn together with T13–T24 T-epitope-containing peptides; n = 36). Experiment B: group 1 (untreated control group; n = 6), group 2 (ADAD and synthetic immunomodulator AA0029 [ADADs]; n = 6), groups 3–14 (ADADs together with B1–B12 B-epitope-containing peptides; n = 36) and groups 15–26 (ADADs together with T13–T24 T-epitope-containing peptides; n = 36). The mice were subcutaneously immunised using an adjuvant adaptation (ADAD) system [23].

Briefly, the ADAD vaccination system included the vaccine antigen, an immunomodulator (natural or chemically-synthesised), together with non-haemolytic adjuvant *Quillaja saponaria* (QS) saponins to form an emulsion with non-mineral oil in a 70/30 oil/water ratio. Vaccination with this system included a set of 2 subcutaneous injections. The first, also called adaptation, contained QS and the immunomodulator emulsified in non-mineral oil, but without the vaccine antigen; the second injection was administered 5 days after adaptation and contained the vaccine antigen with QS and the immunodulator in the emulsion oil. The individual doses injected during mice immunisation were formulated as follows: 600 µg natural immunodulator PAL (ASAC Pharmaceutical International, Alicante, Spain) or 100 µg chemically synthesised aliphatic diamine immunomodulator AA0029 [24] together with 20 µg *Q. saponaria* (QS) and, when evaluated, 10 µg of each peptide. A final 100 µL/injection volume was emulsified with non-mineral oil (Montanide ISA763A, SEPPIC, Paris, France) in a 70/30 oil/water ratio. Mice were immunised on day 0 and two booster doses with 100 µL of the preparations mentioned above were administered on days 14 and 28.

### Sample collection

Mice were humanely euthanised 2 weeks after third immunisation. The spleen was aseptically removed during necropsy to obtain splenocytes for *in vitro* assays. Spleen cell suspensions were collected by spleen perfusion by passing sterile phosphate buffered solution (PBS) through individual spleens according to the methodology described elsewhere by [31]. Blood samples for each mouse were obtained before each immunisation and also during necropsy for serological studies.

### Measuring antibody responses

Sera from mice immunised with the aforementioned formulations were analysed by ELISA for measuring total IgG, IgE and IgM levels as well as IgG1 and IgG2a antibody isotype levels. Briefly, 96-well polystyrene microplates (Costar, Corning Costar Corp, Cambridge, Mass) were coated with 1 µg of each peptide in carbonate buffer pH 9.6 (100 µL per well) and incubated overnight at 4°C. The plates were then washed thrice for 5 min with PBS containing 0.05% Tween 20 (PBST). The plates were blocked with 5% skimmed milk (SM) in PBST (200 µL per well) for 1 h at 37°C and then washed again thrice, as described above. Sera samples were appropriately diluted at 1∶100 in dilution buffer (5% SM and PBST) and added to the wells (100 µL per well) in duplicate. After 1 h incubation at 37°C the plates were washed as described above and, according to each assay, goat peroxidase-conjugated anti-mouse IgG, IgE, IgM, IgG1 and IgG2a (1∶1,000 in dilution buffer, 100 µL per well; Sigma) were incubated for 1 h at 37°C. After washing as above, the bound antibodies were detected using H_2_O_2_ (0.012%) and ortho-phenylenediamine (0.04%) in 0.1 M citrate/phosphate buffer (100 µL per well). The enzyme reaction was stopped after 15–20 min by adding 3N H_2_SO_4_ (100 µL per well) and optical density was measured at 550 nm (OD_550_) on an Ear400FT ELISA reader (STL Lab Instruments, Groding, Austria). The mean absorbance values for each mouse serum from each group were determined and included in each data point.

### Cytokine determination

The frequencies of antigen specific IFN-γ, IL-1α, IL-2, IL-4, IL-5, IL-6, IL-10, IL-17, TNF-α producing T-cells in the spleens were quantified by using a flow cytometry-based methodology. Individual mouse splenocytes were cultured in 6-well plates at 1×10^6^ cells per well concentration in complete medium (RPMI 1640 medium containing 10% heat-inactivated foetal bovine serum and antibiotics, 100 U/mL penicillin and 100 µg/mL streptomycin) and stimulated with each synthetic peptide at final 10 µg/mL concentration for 72 h at 37°C in a humidified atmosphere with 5% CO_2._ Control wells were prepared containing untreated mouse splenocytes. After the incubation period, splenocyte culture supernatants were recovered for cytokine determination. A FlowCytomix Mouse Th1/Th2 10plex kit (Bender MedSystems GmbH, Vienna, Austria) was used, according to the manufacturer’s instructions. Briefly, different sized fluorescent beads coated with capture antibodies specific for the cytokines mentioned above were incubated with splenocyte supernatant cell culture samples to form sandwich complexes with phycoerythrin (PE)-conjugated secondary antibodies. Flow cytometry data were collected using a FACSCalibur flow cytometer (BD Biosciences) at the University of Salamanca’s Flow Cytometry Central Service. A total of 8,000 events were collected gated by forward and side scatter and data were analysed using FlowCytomix Pro 3.0 software (Bender MedSystems, Vienna, Austria). Each cytokine concentration was determined from standard curves using known concentrations of mouse recombinant cytokines.

### Experimental identification of linear B- and T-cell epitopes

An ELISA assay was used for evaluating the presence of each B-cell epitope using sera from infected mice. Briefly, plates were coated with 1 µg of either each synthetic peptide or *F. hepatica* excretory/secretory antigen and incubated overnight at 4°C. After blocking and washing steps, as previously described, sera samples from mice infected with *F. hepatica* metacercariae or immunised with each synthetic peptide were appropriately diluted at 1∶100 dilution and added to the wells in duplicate. Serial dilutions of sera from infected mice were made to calculate the specific titre. The plates were washed after 1 h incubation at 37°C and goat peroxidase-conjugate anti-mouse IgG added to the plates (1∶1,000 in dilution buffer, 100 µL per well; Sigma) which were then incubated for 1 h at 37°C. After washing as above, bound antibodies were detected using H_2_O_2_ (0.012%) and ortho-phenylenediamine (0.04%) in 0.1 M citrate/phosphate buffer (100 µL per well).

A flow cytometry analysis was performed on stained and fixed splenocytes to investigate T-cell populations responding to the immunisation of mice with T-cell epitope-containing peptides. Regarding immunofluorescence staining, 5×10^5^ cells were incubated with fluorescein isothyosanate (FITC)-conjugated mouse monoclonal antibodies (mAb) against CD4, or with phycoerythrin (PE)-conjugated mouse mAb against CD8, or with allophycocyanin (APC)-conjugated mouse mAb against CD62L. All samples were incubated with anti CD16/CD32 blocking monoclonal antibody for 5 min at room temperature. Each specific antibody (BD Biosystems) was incubated in 1/50 dilution factor in PBS plus 2% foetal calf serum (PBS-FCS) for 30 min at 4°C. The cells were washed with PBS-FCS after the incubation period, spun at 1,200 rpm for 5 min and the supernatant discarded. Splenocytes were fixed with 100 µL of a solution containing 2% p-formaldehyde in PBS for no longer than 12 h at 4°C before data acquisition. Data was collected using a FACSCalibur flow cytometer (BD Biosciences) at the University of Salamanca’s Flow Cytometry Central Service. A total of 30,000 events were collected (gated by forward and side scatter) and data were analysed using Gatelogic Flow Cytometry Analysis Software (Inivai Technologies Pty Ltd).

### *In vivo* protection studies: antigens, vaccine formulation and immunisation trials

Based on the immune response induced by each of the peptides assayed, those inducing the following immunological patterns were selected for the *in vivo* protection studies: peptides inducing a high -but not unique- Th1 response; peptides inducing a strong Th2 response; peptides inducing a Treg response, peptides inducing a mixed Th1/Th2 response and peptides inducing a combination of Th1/Th2/Treg/Th17 response. The aforementioned peptides were selected as vaccine candidates due to their high immunogenicity, but taking also into account that immune mechanisms associated with protection against *F. hepatica* are not well understood. Concerning the immunomodulator, the one inducing the highest overall immune response was selected. Seventy mice were divided into 10 groups as follows: group 1 consisted of untreated and uninfected controls, group 2 untreated and infected controls, group 3 adjuvant-administered and infected controls, group 4 those immunised with peptide B1, group 5 mice immunised with peptide B2, group 6 immunised with peptide B5, group 7 immunised with peptide B6, group 8 immunised with peptide T14, group 9 immunised with peptide T15 and group 10 immunised with peptide T16. The mice were subcutaneously immunised using the adjuvant adaptation (ADAD) system as previously described.

### Experimental infection and protection assessment

All the animals included in this study (except untreated controls) were orally infected with 7 *F. hepatica* metacercariae two weeks after the last immunisation. *F. hepatica* metacercariae were provided by Ridgeway Research Ltd (Gloucestershire, U. K.) and were stored at 4°C on 0.4% carboxymethylcellulose until use. Metacercariae viability was confirmed by microscope observation before infection. Human endpoints were used when an evidence of severe pain, excessive distress, suffering or an impending death was observable in any of the animals and then euthanised with an intraperitoneal injection of pentobarbital at 60 mg/kg using 30 g needles. On day 42 post infection mice remaining alive were then humanely euthanised and necropsied to recover flukes from liver, score hepatic damage and study the survival rates. Mice welfare was evaluated daily according to behaviour and appearance, physiological indicators and other general clinical signs. Two experienced pathologists independently evaluated liver lesions without knowing which group the livers belonged to. Changes regarding size, colour, consistency concerning blood vessels, bile ducts and surface wounds were evaluated; a score was assigned to each feature: 0 points if no lesions was observed, 1 point if a liver lobe was affected, 2 if an entire lobe was affected and 3 if more than 1 lobe was affected. No lesions was assigned when the sum was 0 points, mild (+) for 1–5 points, moderate (++) for 6–10 points and severe (+++) for 11–14 points. Survival rate percentage in mice infected was then calculated as the ratio of the number of surviving experimental mice on day 42 and the total of experimental mice in each group.

### Statistical analysis

An initial descriptive analysis was made of each group of mice, exploring cytokine and immunoglobulin patterns and cell expression. A linear model with interaction was used to evaluate cytokine and cell expression, including two factors: epitope type (B or T) and the immunomodulator used (AA0029 or PAL). Interactions were plotted for representing this linear model using Plotrix package in R. Furthermore, groups of peptides were compared using a Kruskall Wallis test followed by a Dunn multiple comparisons test. Differences having p<0.05 were considered statistically significant. The results were reported as the mean of each group and the standard deviation (SD). SPSS 20.0 software (SPSS Inc., USA) was used for data analysis. Hierarchical clustering was used to identify sets of cytokines whose expression levels correlated among peptides with B- and T-cell epitopes. Centring and scaling are the previous data transformation steps. The complete linkage clustering method was used, based on a similarity matrix derived from Pearson (rows) and Spearman (columns) moment correlations. The heatmap was visualised using the heatmap.2 function of gplots package in R. Kaplan-Meier survival curves were used for evaluating survival rates.

## Results

### Selecting candidate proteins, epitope prediction and peptide synthesis

A total of 269 reported *F. hepatica* protein sequences were accessible at and downloaded from NCBI; these proteins were individually predicted for both SP and TM domains for selecting those likely to be secreted. According to such criteria, 21 proteins were finally chosen, aligned and grouped into six different families. Most were found to be cathepsins and cathepsin-like proteases. Once selected, the proteins were then analysed for determining B- and T-cell epitopes. Twenty-four linear peptides were chemically synthesised according to *in silico* epitope prediction: twelve 20-amino-acid-long peptides for B-cell epitopes (peptides B1 to B12) and twelve 15-amino-acid-long peptides for T-cell epitopes (peptides T13 to T24). Four peptide pairs (B1–B2, B3–B4, B7–B8 and B11–B12) were synthesised in which a cysteine residue was replaced by a threonine in one peptide of the pair. Taking into account that peptides used for immunisation were synthesised adding a glycine and a cysteine at both termini and then polymerised inducing the formation of disulphide bridges, the presence of any additional cysteine residue in a monomer-synthesised peptide could lead to several polymerised forms being obtained from the peptide. To avoid this issue, and to obtain a well-defined polymerised form, we replaced all cysteine residues in the peptide sequence by threonine ones (a residue sharing similar biochemical properties). More than 5 mg of each purified peptide was obtained and its purity was found to be higher than 90%. Table 1 shows the amino acid sequence of each B- or T-cell epitope, indicating which proteins belonged to each peptide.

**Table 1:** Selected *F. hepatica* proteins containing signal peptide (SP) but no transmembrane domain (TM).

Epitope	Synthesised peptide sequence	Position	GenBank	Description	Proteinlength (aa)
B1	KGAGSSQDACIKFIQYEVDG	63–82	AAB02579.1	Amoebapore homologue	102
B2	KGAGSSQDATIKFIQYEVDG	63–82	AAB02579.1	Amoebapore homologue	102
B3	FASFDVPSKQPTIDIDLCDI	14–33	AAF88069.1	Amoebapore-like protein	101
B4	FASFDVPSKQPTIDIDLTDI	14–33	AAF88069.1	Amoebapore-like protein	101
B5	ISEIRDQSSTSSTWAVSSAS	102–121	ABU62951.1	Cathepsin B	337
B6	GVENGVKYWLIANSWNEGWG	293–312	ABU62951.1	Cathepsin B	337
B7	QTCSPLRVNHAVLAVGYGTQ	260–279	AAB41670.2	Secreted cathepsin L1	326
		260–279	AAA29136.1	Cathepsin	326
		264–283	AAP49831.1	Cathepsin L	326
		260–279	Q24940.1	Cathepsin L-like proteinase	326
B8	QTTSPLRVNHAVLAVGYGTQ	260–279	AAB41670.2	Secreted cathepsin L1	326
		260–279	AAA29136.1	Cathepsin	326
		264–283	AAP49831.1	Cathepsin L	326
		260–279	Q24940.1	Cathepsin L-like proteinase	326
B9	YTEPRSVTPEERSVFQPMIL	27–46	AAV68752.1	Cystatin	116
B10	FVPLYSSKSATSVGTPTRVS	95–114	AAV68752.1	Cystatin	116
B11	VTTNGPPNGKHNDKHTYVEC	350–369	CAC85636	Legumain-like	419
B12	VTTNGPPNGKHNDKHTYVET	350–369	CAC85636	Legumain-like	419
T13	TVNLVKRLLQNSVVE	37–51	AAB02579.1	Amoebapore homologue	102
T14	DYIIDHVDQHNATEI	80–94	AAF88069.1	Amoebapore-like protein	101
T15	DRNTQRQTVRYSVSE	69–83	ABU62925.1	Cathepsin B	337
T16	FYMFEDFLVYKSGIY	260–274	ABU62925.1	Cathepsin B	337
T17	KYLTEMSRASDILSH	83–97	AAB41670.2	Secreted cathepsin L1	326
		83–97	ABQ95351.1	Secreted cathepsin L2	326
		83–97	AAA29136.1	Cathepsin	326
		83–97	AAP49831.1	Cathepsin L	326
		83–97	AAR99518.1	Cathepsin L protein	326
		83–97	Q24940.1	Cathepsin L-like proteinase	326
T18	ISFSEQQLVDTSGPW	153–167	AAB41670.2	Secreted cathepsin L1	326
		153–167	AAA29136.1	Cathepsin	326
		153–167	AAP49831.1	Cathepsin L	326
		153–167	BAA23743.1	Cathepsin L	325
		153–167	AAR99518.1	Cathepsin L protein	326
		153–167	AAT76664.1	Cathepsin L1 proteinase	326
		153–167	Q24940.1	Cathepsin L-like proteinase	326
T19	ENAYEYLKHNGLETE	178–192	AAC47721.1	Secreted cathepsin L2	326
		178–192	ABN50361.2	Cathepsin L	326
		178–192	CAA80446.1	Cathepsin L-like protease	326
		53–67	CAA80445.1	Cathepsin L-like protease	166
T20	LDPYFNLVSPEVYNY	29–43	BAE44988.1	Cytochrome oxidase subunit I	145
		29–43	BAE45005.1	Cytochrome oxidase subunit I	145
T21	DLNLPRLNALSAWLL	76–90	BAE44988.1	Cytochrome oxidase subunit I	145
		76–90	BAE45005.1	Cytochrome oxidase subunit I	145
T22	FAGHGKAYLHGSFDK	56–70	AAA29144.1	Vitelline protein B2	272
T23	YEKYEDDYARETPYD	254–268	AAA29144.1	Vitelline protein B2	272
		254–268	AAA29143.1	Vitelline protein B1	272
T24		101–115	AAA31753.2	NADH dehydrogenase subunit 3	118
		101–115	Q34522.1	NADH dehydrogenase subunit 3	118
		101–115	AAG13152.2	NADH dehydrogenase subunit 3	118

GenBank accession number and amino acid sequences from each B- and T-cell chemically synthesised epitope are also indicated.

### *In vitro* peptides cytotoxicity evaluation

Each peptide was assayed in four different concentrations ranging from 1 to 100 µg/mL for *in vitro* cytotoxicity evaluation. Some peptides’ hydrophobic nature resulted in them having low solubility in PBS buffer. Such peptides needed DMSO to be added at a concentration no higher than 2%. Controls for checking solvent cytotoxicity were added to the study (data not shown). Results showed that none of the peptides were toxic for macrophages below 50 µg/mL and more than 90% were still viable after treatment (*Fig. S1*). Some T-peptides showed decreased cell viability at the highest concentration (100 µg/mL) and only 80% of the macrophage population was viable. However, the maximum peptide concentration reached in *in vivo* immunisation assays was no higher than 10 µg/mL.

### The effect on immune response induced by immunising mice with synthetic peptides: antibody response and cytokine levels

**Figure 1:**
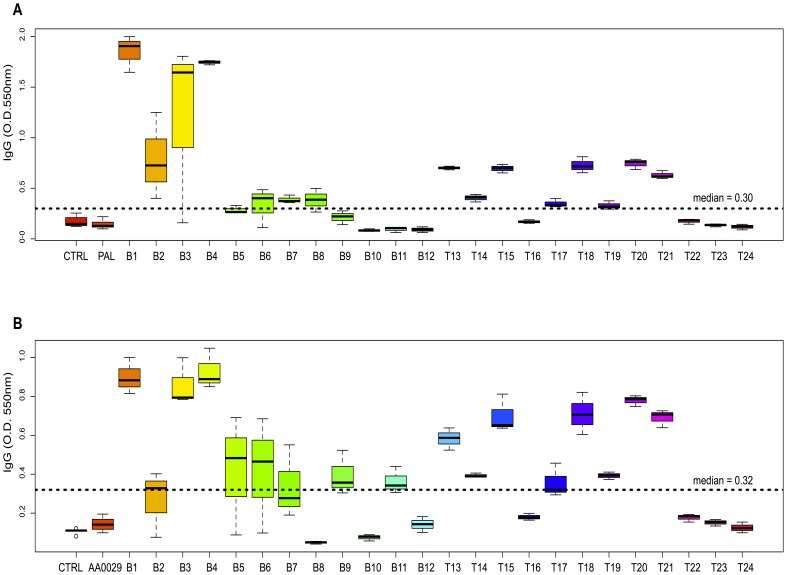
Boxplot showing IgG anti-peptide antibody levels in mice immunised with the synthetic peptides formulated in the ADAD vaccination system. The bottom and the top of the box indicate the 25^th^ and 75^th^ percentiles, respectively. A). Mice immunised using PAL. B). Mice immunised using AA0029.

**Figure 2:**
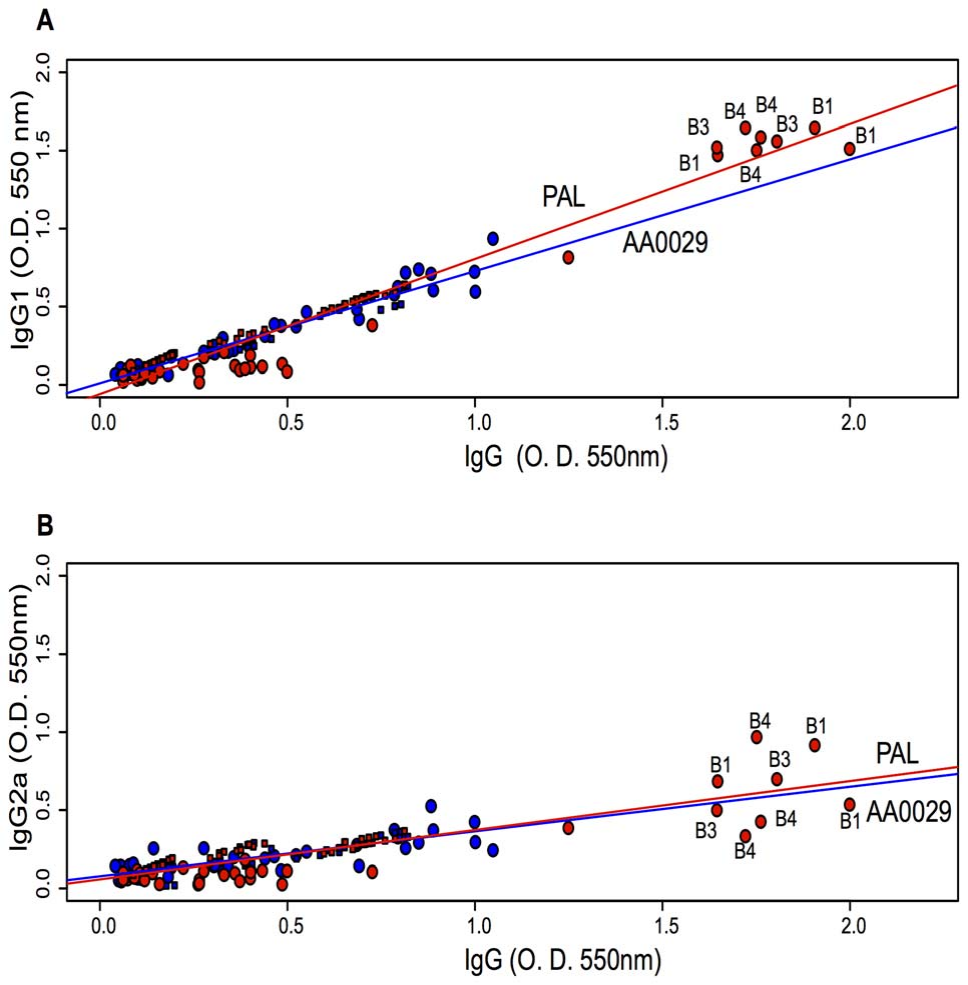
Linear regression comparing the effect of PAL and AA0029 immunomodulators on IgG subtype levels. A). IgG1 related to IgG. B). IgG2a related to IgG. Red indicates the PAL immunomodulator and blue AA0029. Circles and squares represent B- and T-peptides, respectively.

Immunomodulator (PAL and AA0029), epitope (B or T) and peptide effect on antibody levels were studied. Regarding the immunomodulator effect, it was observed that using PAL induced higher IgG levels than AA0029 ([0.5267] *cf* [0.4332] p = 0.003). Significantly higher total IgG levels were obtained when B-epitope-containing peptides were used compared to T-epitope-containing peptides, regardless of adjuvant effect ([0.5258] *cf* [0.4332] p = 0.05). The boxplot in *Fig. 1A*, showed that peptides B1, B2, B3, B4, T18 and T20 induced the highest levels of IgG after being administered in mice using the PAL compared to the control group (p<0.05), whilst peptides B1, B3, B4, B5, B6, T13, T15, T18, T20 and T21 induced the highest levels after being injected using the AA0029 compared to the control group (p<0.05) (*Fig. 1B*). Concerning those peptides with similar amino acid sequences (different in one amino acid only, where a cysteine was replaced by a threonine), significant differences in IgG levels were only observed in the B1–B2 peptide pair, being the peptide B1 more immunogenic than peptide B2 when using any of the immunomodulators. Regarding IgG subclasses, the following relationships were described through linear regression adjustment: IgG1 with total IgG (*Fig. 2A*) and IgG2a with total IgG (*Fig. 2B*). It was observed that PAL induced significantly higher IgG1 levels than AA0029 (p<0.05) in immunised mice (*Fig. 2A*). IgG2a levels were not influenced by the immunomodulator used, since there were no statistical differences between the slopes (*Fig. 2B*). Peptides B1, B3 and B4 induced the highest IgG1 and IgG2a levels with the PAL immunomodulator.

There were no statistical differences regarding IgE levels when either of the immunomodulators was used. Furthermore, B-epitope-containing peptides showed a stronger IgE response than T-epitope-containing peptides ([0.1588] *cf* [0.0952] p<0.01). The same pattern was observed in anti-peptide IgM antibodies, as there were no statistical differences when comparing immunomodulator effect; however, B-epitope-containing peptides had a stronger response than T-epitope-containing ones ([0.1161] *cf* [0.1014] p<0.05). The boxplot showing the IgE and IgM anti-peptide for each peptide has been included in *Figs. S2 & S3*.

The type of immunomodulator and epitope used in the immunisation trial influenced cytokine levels, as can be seen in the three-dimensional scatterplots in *Figs S4 & S5*. Specifically, using AA0029 with B-epitope-containing peptides led to obtaining high levels of IFN-γ, IL-4, IL-10 and IL-17, whilst using peptides containing T-epitopes and the AA0029 immunomodulator produced higher IFN-γ, IL-4 and IL-17 levels. Changes in cytokine level may thus have resulted from the interaction between the immunomodulator (PAL or AA0029) and the epitope (B or T) being used (*Fig. S6*).

**Figure 3:**
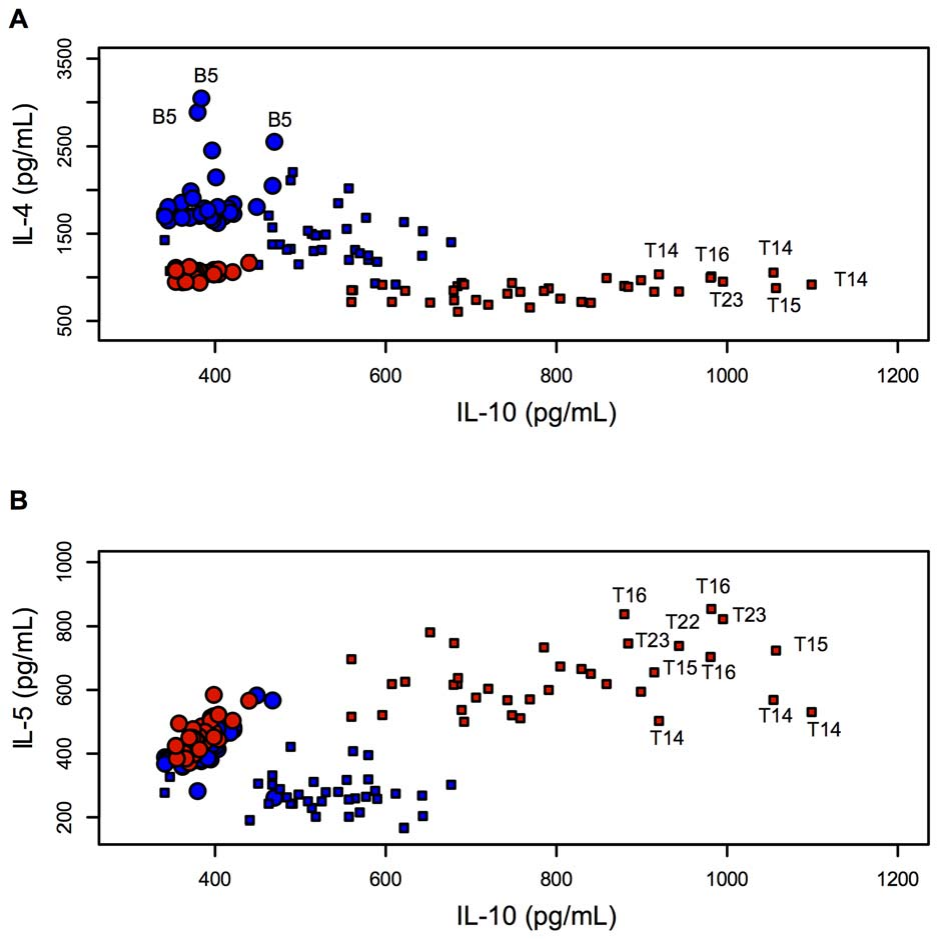
Scatterplot comparing the effect of PAL and AA0029 immunomodulators on cytokine levels. A). IL-4 related to IL-10. B). IL-5 related to IL-10. Red indicates PAL and blue AA0029. Circles and squares represent B- and T-peptides, respectively.

**Figure 4:**
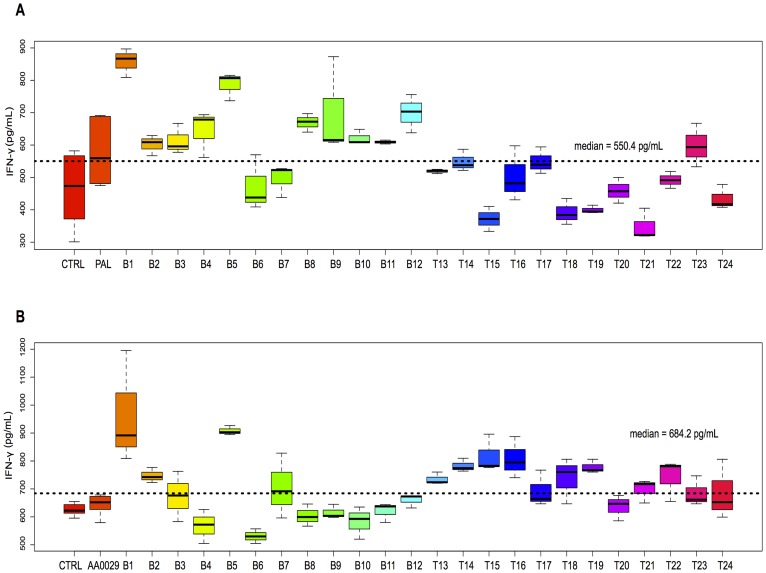
Boxplot showing IFN-γ cytokine levels in splenocyte mouse cell culture immunised with synthetic peptides. A). Mice immunised with PAL. B). Mice immunised with AA0029.

**Figure 5:**
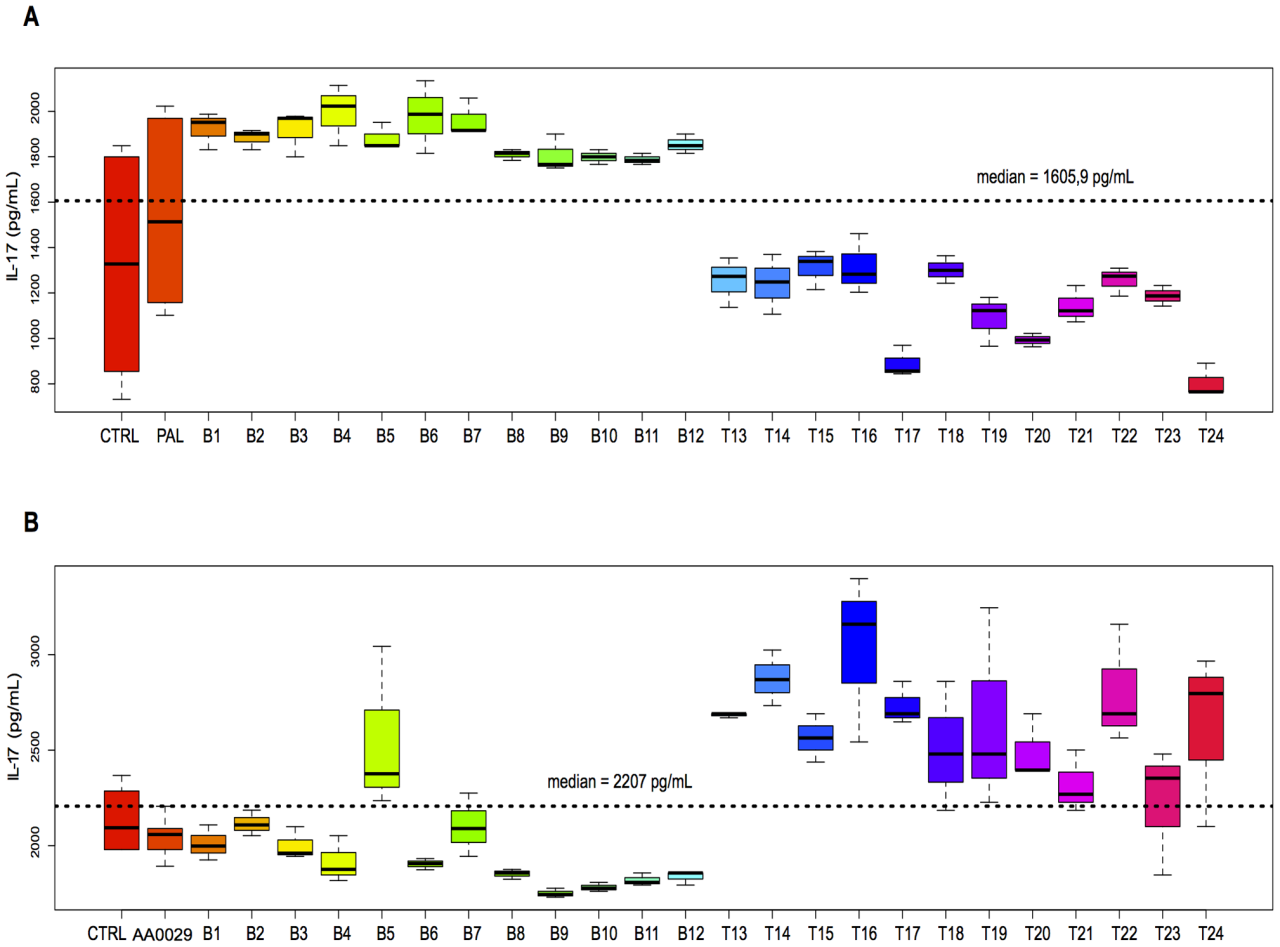
Boxplot showing IL-17 cytokine levels in splenocyte mouse cell culture immunised with synthetic peptides. A). Mice immunised with PAL. B). Mice immunised with AA0029.

Each peptide was also individually analysed for cytokine quantitation after being injected into the mice using the ADAD vaccination system. The following relationships were described: IL-4 and IL-10 levels (*Fig. 3A*) and IL-5 and IL-10 levels (*Fig. 3B*) in all the mice used in this study. Figure 3A and *Fig. 3B* show that most T-epitope-containing peptides induced high IL-10 and IL-5 levels, but lower IL-4 levels. Increased IL-4 levels were observed in mice immunised with peptides B5, B6, T14 and T15 compared to the AA0029 adjuvant-vaccinated group and also peptides B6 and B12 when the PAL immunomodulator was used. Peptides T16, T22 and T14 induced changes in IL-10 levels when these peptides were formulated with AA0029, whilst T15, T16, T22 and T23 did so with PAL. Concerning IL-5 levels, peptides B1, B2, B3, B4, B6, B7 and T17 produced the highest IL-5 levels with AA0029, whereas peptides T13, T15, T16, T17 and T20 did so with PAL. Regarding Th1 cytokine profile, the boxplot in *Fig. 4* shows IFN-γ levels; these became increased for peptides B1, B5, T15 and T16 compared to the AA0029 adjuvant-vaccinated group, whilst peptides B1, B5, B8, B9 and B12 produced changes in IFN-γ when PAL was used. Concerning peptides with similar amino acid sequence (Cys by Thr substitutions), higher IFN-γ levels were obtained with peptide B1 (compared to peptide B2), using either immunomodulator. Peptide B8 also induced higher levels of IFN-γ than peptide B7 but only when using the PAL immunomodulator. Changes in IL-17 levels were produced by peptides T14, T16 and T22 when AA0029 was used and by peptides B1, B3, B4, B6 and B7 when using PAL (*Fig. 5*). These results clearly showed that some peptides stimulated the production of cytokines associated with different T-helper cell profiles. It has been observed that the AA0029 immunomodulator has induced a more powerful, but not unique, Th2-like immune response. B-peptides only had a Th1-type immune response for peptide B1 whilst T-peptides elicited a mixed Th1/Th2 immune response when the natural PAL immunomodulator was used.

**Figure 6:**
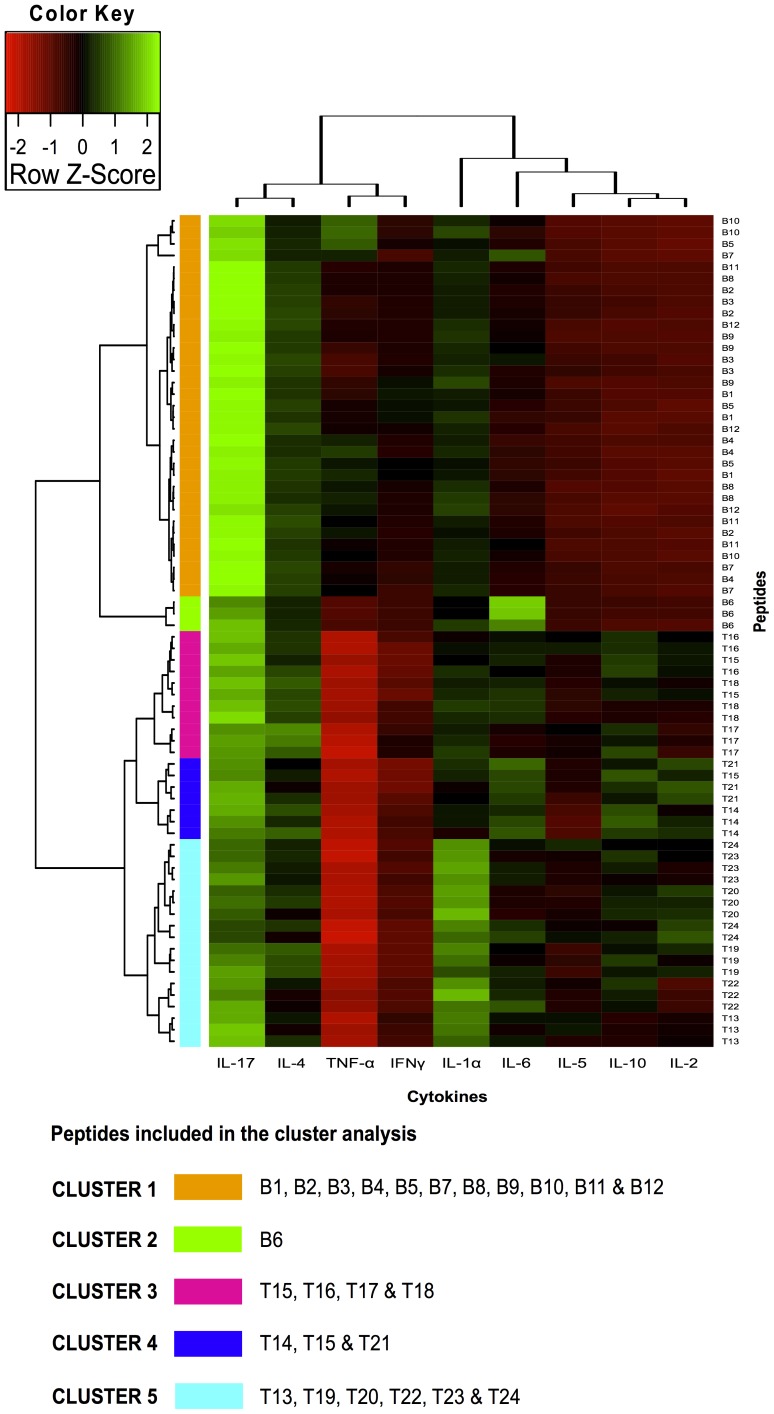
Individual cytokine expression levels are represented by shades of green to red in the central heatmap (highest values shown in green and lowest in dark red). The right-hand margin provides the names of peptide sets. Rows and columns represent clusters of interleukins and peptides having a similar immunological response. A list of cytokines grouped within each cluster is also provided. Groupings having shorter distances (as indicated by the distance to k-means nearest group) had greater similarity. Bicluster analysis for B- and T-cell peptides formulated with the PAL immunomodulator. Five major clusters can be discerned (1, 2, 3, 4 and 5), encompassing peptides having similar cytokine levels.

**Figure 7:**
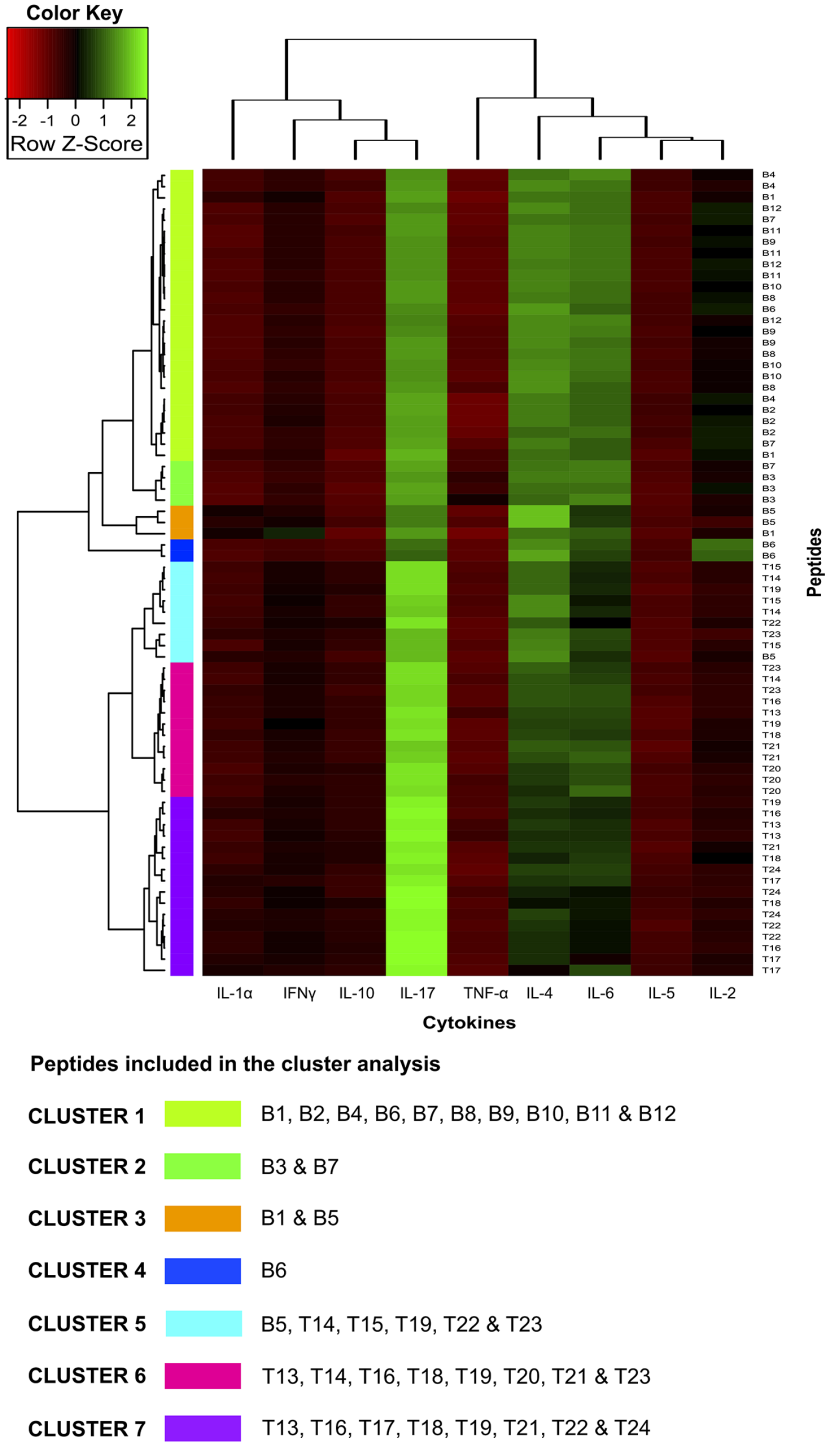
Individual cytokine expression levels are represented by shades of green to red on the central heatmap (highest values shown in green and lowest in dark red). The right-hand margin provides the name of peptide sets. Rows and columns represent clusters of interleukins and peptides having a similar immunological response. A list of cytokines grouped within each cluster is also provided. Groupings having shorter distances (as indicated by the distance to k-means nearest group) had greater similarity. Bicluster analysis for B- and T-cell peptides formulated with the AA0029 immunomodulator. Seven major clusters can be discerned (1, 2, 3, 4, 5, 6 and 7), encompassing peptides having similar cytokine levels.

Although analysing individual cytokines identified peptides associated with different cytokine level patterns, such associations did not represent the relationship between the peptides or cytokines included in this study. Two-dimensional cluster analysis was therefore performed to identify sets of cytokines that might have been coordinately expressed induced by immunisation with peptides having a low immune response (compared to a high one) and more strongly correlate them with an effective immune response. Bicluster analysis led to a comprehensive representation of splenocyte state throughout their response to peptides used in this study. The expressed cytokines’ functional concordance gave biological significance to the broad patterns seen in images like the biclusters in *Figs. 6* *&* *7* for PAL and AA0029, respectively. Peptides containing B- and T-cell epitopes clustered separately: cluster 1–2 and cluster 3–5 for the PAL adjuvant (*Fig. 6*) and cluster 1–4 and cluster 5–7 for adjuvant AA0029 (*Fig. 7*). It was also observed that cytokine correlation depended on the adjuvant used in vaccine formulation. PAL-adjuvant formulation clustered IL-17, IL-4, IFN-γ and TNF-α separately from IL-1α, IL-6, IL-5, IL-10 and IL-2 cytokines. AA0029-adjuvant formulations induced different cytokine co-expression pattern; IL-1α, IFN-γ, IL-10 and IL-17 clustered separately from TNF-α, IL-4, IL-6, IL-5 and IL-2. This result confirmed the strong immunomodulator effect conferred by the adjuvant being used.

Analysis of B-epitopes clustered with the PAL adjuvant (*Fig. 6*) revealed that cluster 1 was able to cluster each B-epitope-containing peptide, except peptide B6, while cluster 2 represented the most interesting cluster in *Fig. 6* because it clustered the three B6 peptide replicates which induced high IL-4, IL-6 and IL-1α levels. Regarding B-peptides clustered with AA0029 (*Fig. 7*), a single cluster (cluster 4) also represented two of the three B6 peptide replicates, which also induced high IL-4 and IL-5 levels. Cluster 3 in *Fig. 7* included peptide B1 that induced high IL-5, IFN-γ and TNF-α levels and peptide B5 that also induced high IL-4 and IFN-γ levels, suggesting different immunological effects induced by such B-peptides regarding the groups that clustered T-epitopes using the PAL adjuvant (*Fig. 7*). Peptides T14, T15 and T16 were included in clusters 3 and 4; they were able to induce high levels of regulatory cytokine IL-10, IL-5 and proinflammatory cytokine IL-2. Regarding T-epitope clustered groups using AA0029 (*Fig. 7*), the peptides were extremely disseminated throughout the different clusters, although T14 and T15 both seemed to group in cluster 5, inducing high IL-4 and IFN-γ levels.

### Experimental identification of linear B- and T-cell epitopes

**Figure 8:**
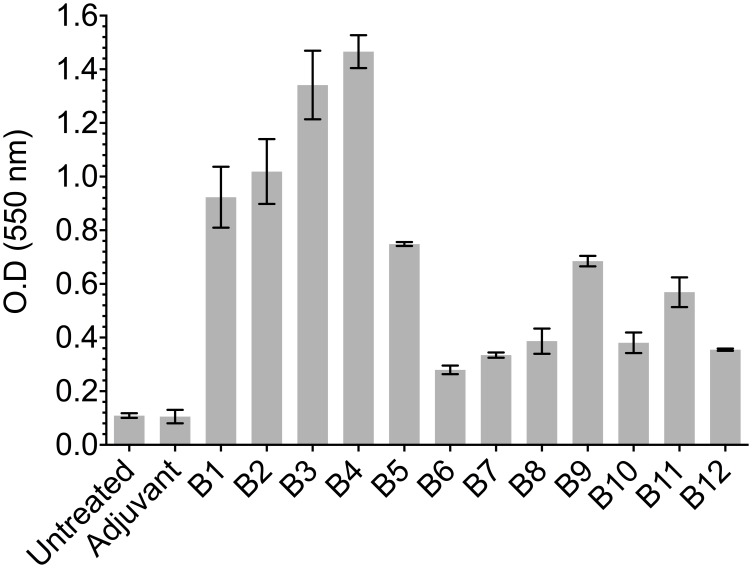
ELISA showing B-cell epitope-containing synthetic peptide reactivity against a pool of sera from mice infected with *F. hepatica* metacercariae.

**Figure 9:**
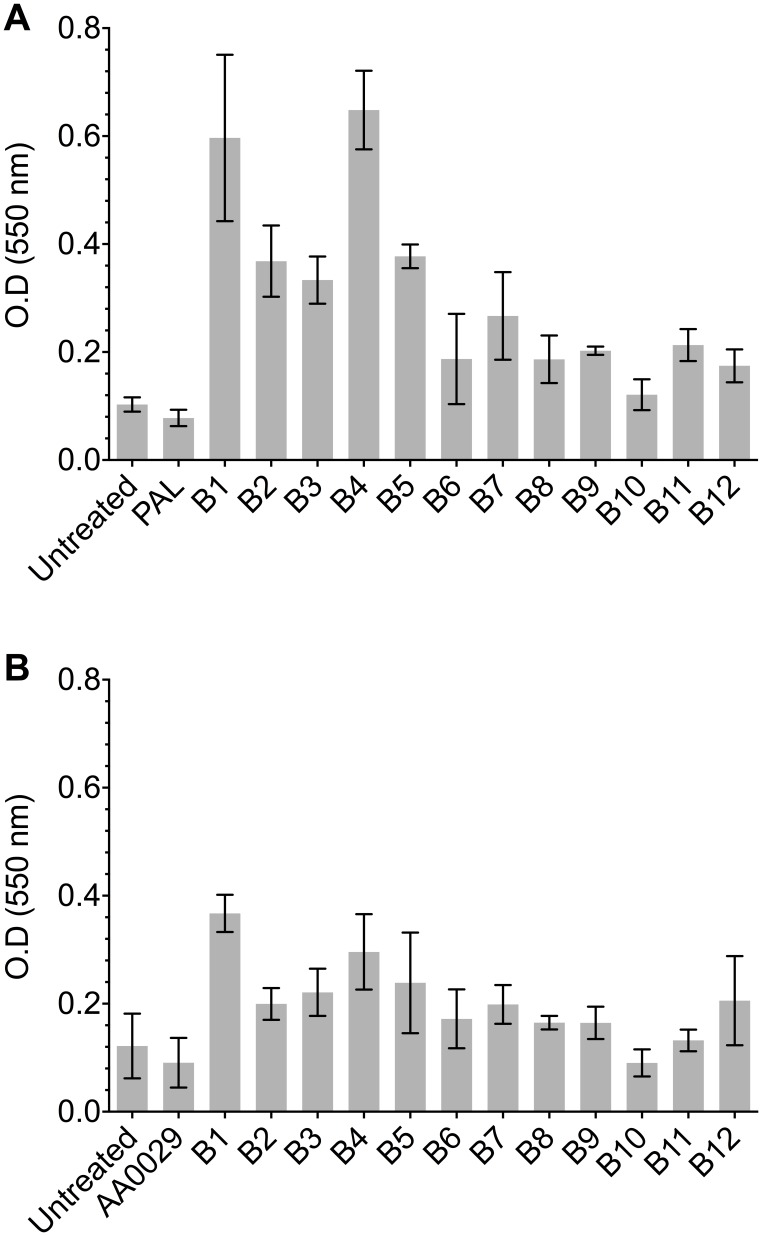
*F. hepatica* excretory/secretory antigen reactivity against hyper-immune sera from mice immunised with synthetic peptides containing B-cell epitopes as assessed by ELISA. A). B-cell peptides formulated with PAL. B). B-cell peptides formulated with AA0029.

An ELISA assay was used for evaluating the recognition of peptides containing predicted B-cell epitopes using sera from mice infected with *F. hepatica*. Figure 8 shows that peptides containing predicted B-cell epitopes had some reactivity against sera from infected mice, thus indicating the presence of linear epitopes within the synthetic peptides. *F. hepatica* amoebapore and cathepsin B protein-derived B1, B2, B3, B4 and B5 peptides had the highest reactivity, reaching 1∶3,200 specific titre values (data not shown). The ELISA depicted in *Fig. 9* shows the reactivity of sera raised against predicted B-cell epitopes formulated in either PAL or AA0029 immunomodulators against *F. hepatica* excretory/secretory antigens. Higher reactivity was observed when using hyper-immune sera from mice immunised with the B-cell epitopes formulated with the PAL immunomodulator (*Fig. 9A*). The highest reactivity was shown by B1, B2, B3, B4 and B5 peptides in both cases, thereby confirming the presence of immunogenic regions within peptides containing predicted B-cell epitopes (*Figs. 9A & 9B*).

**Figure 10:**
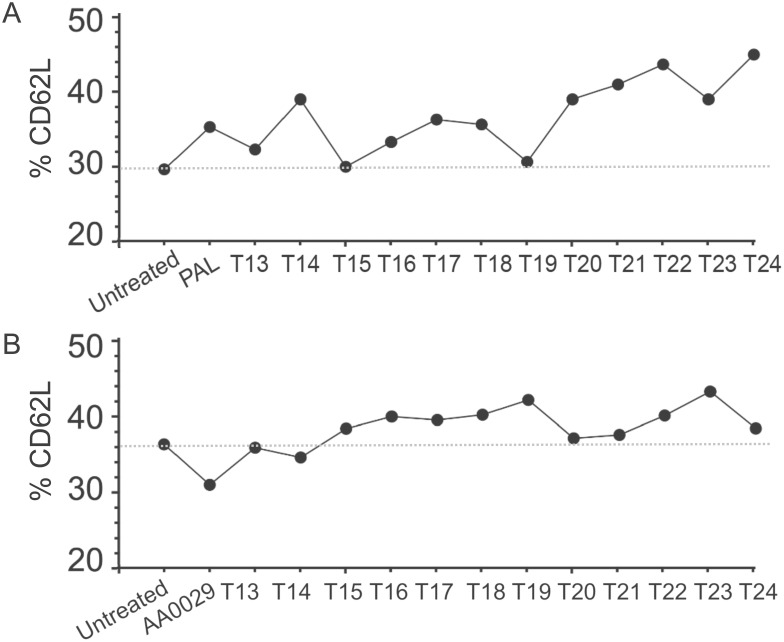
Flow cytometry analysis for quantitation of T-lymphocyte population through CD62L^+^ T-cell memory marker in mice immunised with T-cell epitope-containing synthetic peptides. A). T-cell peptides formulated with PAL. B). T-cell peptides formulated with AA0029.

Three lymphocyte subsets (LT CD4^+^, LT CD8^+^ and LT CD62L^+^) were analysed to better evaluate the cellular immune response of T-cell epitope-containing synthetic peptides formulated with both the PAL and/or AA0029 immunomodulators. No statistically significant differences were detected in either LT CD4^+^ or LT CD8^+^ immunophenotypes in mice immunised with the T-cell epitope-containing peptides. However, differences in the CD62L^+^ immunophenotype were detected in mice immunised with peptides containing T-cell epitopes formulated in either of the immunomodulators (PAL or AA0029) when compared to the non-immunised control group. Different lymphocyte expression patterns were observed when the peptides were individually analysed. Figure 10 shows the LT CD62L^+^ levels reached by each group of mice immunised with T-cell epitope-containing peptides formulated with either the PAL (*Fig. 10A*) or AA0029 immunomodulator (*Fig. 10B*). Using the PAL immunomodulator led to higher LT CD62L^+^ levels being obtained in mice immunised with T-cell epitopes, except those immunised with peptides T13, T15 and T19 (*Fig. 10A*). Concerning the AA0029 immunomodulator, its use led to an increase in the LT CD62L^+^ population in mice immunised with the T-cell epitopes, except those immunised with peptides T13, T14, T20 and T21 (*Fig. 10B*). The results obtained here confirmed the presence of immunodominant T-cell epitopes in peptides selected using a bioinformatics approach.

### *In vivo* protection assessment

**Figure 11:**
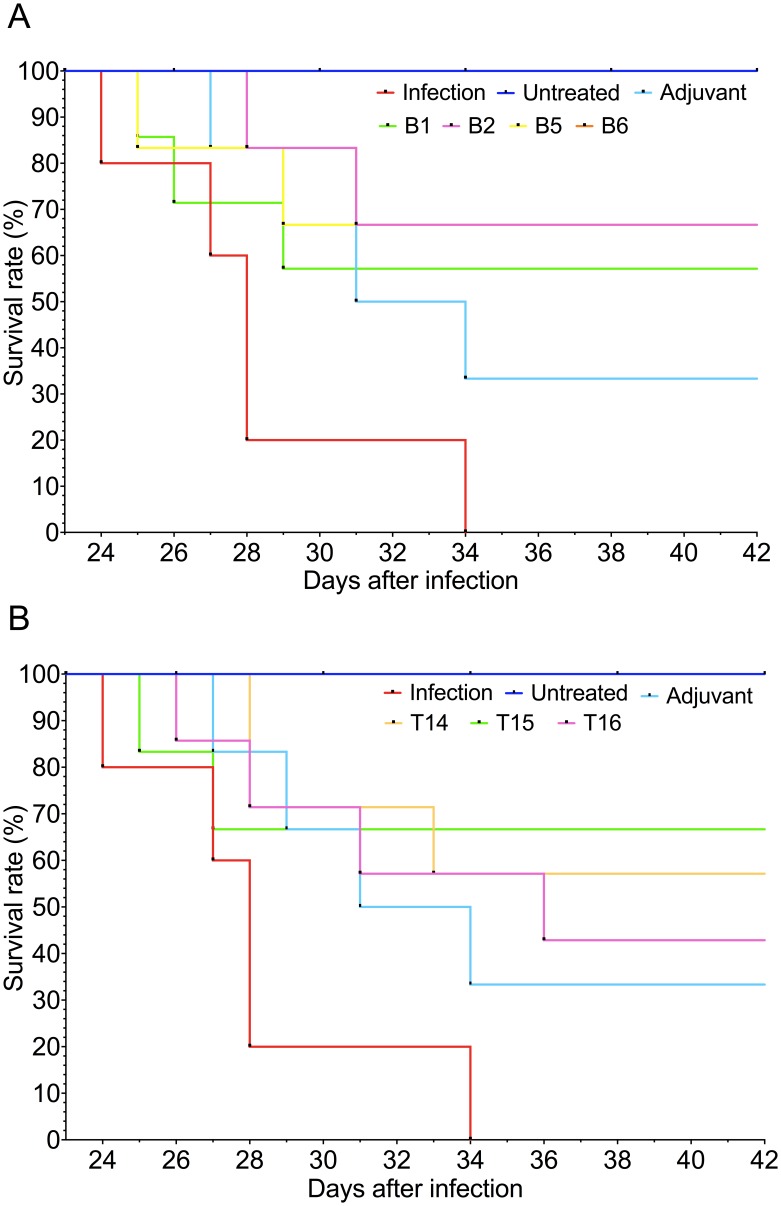
Kaplan-Meier curves depicting survival rates in mice immunised with single peptides and infected with *F. hepatica* metacercariae. A). Mice immunised with single peptides containing B-epitopes. B). Mice immunised with single peptides containing T-epitopes. Survival rates of mice belonging to both untreated and infected controls are also represented. Human endpoint was established when an indicator of severe pain, excessive distress, suffering or an impending death was observed in any of the animals and then euthanised with an intraperitoneal injection of pentobarbital at 60 mg/kg using 30 g needles.

Immunising mice with the synthetic peptides enhanced their survival rate, compared to the unimmunised and infected control group. All mice in the unimmunised and infected control group died between 24 and 34 days pi; mice which died before day 42 pi were considered unprotected in our study. Figure 11 shows that all the groups of mice immunised with the synthetic peptides had some degree of protection against experimental infection with *F. hepatica* metacercariae. The highest survival rate (66.7% in each group) was obtained with the peptides B2, B5, B6 and T15 (groups 5, 6, 7 and 9, respectively), according to Kaplan-Meier estimates (*Figs. 11A & 11B*). Mice immunised with single peptides B1 and T14 (groups 4 and 8, respectively) had a 57.1% survival rate (*Figs. 11A & 11B*) and mice immunised with the single peptide T16 (group 10) had a survival rate of 42.9% (*Fig. 11B* *).*

Immunising mice with the peptide B5 (group 6) produced the lowest fluke burden compared to the unimmunised and infected control group ([0.7±0.3] *cf* [2.0±0.3]) (p<0.05). Mice immunised with the single peptides B2 and T15 decreased worm burden by 58% compared to the unimmunised and infected control group. Concerning liver damage score, mice immunised with the single peptide B5 showed the lowest hepatic damage when compared to the infected control group ([7.3±2.1] *cf* [12.0±01.5]) (p<0.05). Mice immunised with the peptide B2 also reduced the liver damage ([8.0±1.3] *cf* [2.0±0.3]) (p = 0.05). These results suggest a direct correlation between the survival percentage with lower liver fluke burdens and liver damage scores (Table 2).

**Table 2:** Recovered flukes and assessment of macroscopic hepatic lesions in CD1 mice immunised with synthetic peptides containing B- and T-cell epitopes using the ADAD vaccination system and challenged with 7 *F. hepatica* metacercariae.

Group	Treatment	Number of flukes in individual mice	Worm recovery (Mean ± SEM)	Reduction (%)	Hepatic lesion in individual mice	Lesion score (Mean ± SEM)	Reduction (%)
1	Untreated uninfected						
2	Infected	3, 1, 2, 2, 2	2.0±0.3		14, 14, 12, 10, 10	12.0±1.5	
3	Adjuvant treated	0, 2, 0, 2, 2, 0	1.0±0.4	50	10, 14, 5, 11, 14, 2	9.3±1.8	22
4	B1	1, 0, 1, 2, 1, 1, 1	1.0±0.2	50	10, 12, 6, 11, 6, 9, 11	9.3±1.5	23
5	B2	0, 1, 1, 1, 1, 1	0.8±0.2	58	6, 14, 5, 9, 9, 5	8.0±1.3	33
6	B5	1, 0, 1, 0, 2, 0	0.7±0.3	67*	4, 1, 12, 12, 7, 8	7.3±2.1	39*
7	B6	2, 0, 2, 1, 0, 1	1.0±0.3	50	13, 2, 8, 10, 14, 13	10.0±1.7	17
8	T14	0, 1, 2, 1, 1, 0, 1	0.9±0.3	57	4, 7, 10, 13, 14, 9, 10	9.6±1.8	20
9	T15	0, 2, 0, 1, 2, 0	0.8±0.4	58	6, 14, 5, 4, 14, 10	8.8±1.7	26
10	T16	1, 0, 0, 1, 2, 1, 1	0.9±0.3	57	10, 10, 11, 5, 13, 12 14	10.7±1.1	11

*p<0.05 compared to infected controls.

## Discussion

The present study was designed to bioinformatically predict and experimentally assess both the immune response and protection-inducing ability of *F. hepatica* protein-derived peptides containing B- and T-cell epitopes. The newly-emergent discipline of bioinformatics, immunoinformatics and rational vaccine design were used here, involving a reverse vaccinology strategy which has been successfully used in malaria and some helminthic infections [11], [14], [17]–[21]. According to reports in the pertinent literature, cysteine proteases released by *F. hepatica* play a key role in parasite feeding, migration through host tissues and immune evasion and they are considered good vaccine candidates [32]. Cysteine proteases have been used as vaccine candidates in immunisation trials involving experimental models such as cattle and sheep and using different adjuvants, resulting in up to 79% protection [33]–[38]. The rationale behind selecting proteins that could be secreted by *F. hepatica* has been based on the fact that these proteins are exposed to a host’s immune system, making them easily recognisable. Most epitopes included in this study belonged to either the cathepsin L or cathepsin B family, eliciting high activation of the immune response. Cathepsin B is predominantly released during the juvenile life-cycle stage whilst cathepsin L is released throughout the whole cycle, thereby representing an important issue when designing a multi-stage, multi-epitope, subunit-based vaccine against *F. hepatica* [32]. Other studies have also shown the importance of two *F. hepatica* cathepsins (FheCB and FheCL), expressed in the infective stage of newly excysted juveniles (NEJs), in acquiring infection. Silencing the expression of both these proteins by using the interference RNA (iRNA) methodology induced less movement and penetration of NEJs to the gut [39]. The present study has demonstrated that immunisation with peptides containing B- and T-cell epitopes formulated with the AA0029 immunomodulator induced a strong immune response -based on high IL-4 and IFN-γ cytokine levels-, these being important biomarkers in Th2 and Th1 differentiation [40]. This pattern could be essential for using these immunomodulator in vaccination trials where a more powerful Th1 or Th2 immune response would be required for protection against disease. Furthermore, AA0029 was able to stimulate some pro-inflammatory and Th17 cytokines, such as IL-6 and IL-17, while PAL previously tested in other immunisation experiments was associated with a down-regulated Th2 immune response [31]. PAL, together with T-epitopes, was able to induce high IL-10 and IL-5 cytokine levels and PAL plus B-epitopes seemed to better stimulate IgG1, the antibody subtype classically belonging to the Th2 immune response profile. However, other components in the ADAD vaccination system used here could modify or highly modulate the immune response, such as the adjuvant Qs21 and the non-mineral oil Montanide. These components could increase antigen immunogenicity, could be used to enhance immune response speed and duration and could stimulate cell-mediated immunity [41]. According to reports in the pertinent literature, Qs21 acts as an immunostimulatory adjuvant involved in Th1 cytokine (i.e. IL-2 and IFN-γ) and IgG2a isotype antibody induction [42]. It is well-known that cattle are highly susceptible to primary infection but are resistant to reinfection, thus generating chronically and silent *F. hepatica* infection [43]. The mechanisms associated with such tolerance are mediated by host’s humoral and cellular Th2 immune responses.

A prolonged Th2 immune response to *F. hepatica* infection is thus not protective and can lead to the parasite residing within the liver or passageways conducting bile. According to the literature, the mechanism behind chronic *F. hepatica* infection development involves IgG1 antibody generation and little or no IgG2a [44], [45]. However, an antibody response against *F. hepatica* cathepsin L as vaccine candidate involves generating both IgG1 and IgG2a antibodies, suggesting a mixed Th1/Th2 protective immune response [34], [44]. The results obtained here showed that most peptides produced both IgG1 and IgG2a antibodies (having higher IgG1 titres in all cases), showing a mixed Th1/Th2 immune response, whilst induced IgE and IgM immunoglobulin levels resulted in very low levels for all the synthetic peptides used in this study. Helminth infection has been associated with IgE response; however, its role in protective immunity is not well understood [46]. There is some evidence for a positive correlation between IgE level and protection in human populations infected with *Schistosoma mansoni* and *Schistosoma haematobium* [47]. Despite the low IgE levels obtained by immunisation with synthetic peptides in the present study, some peptides were able to induce higher levels compared to the adjuvant-vaccinated group.

Cytokine quantitation in stimulated-splenocyte supernatant cell cultures has indicated that B- or T-cell epitope-containing peptides have been able to induce high levels of Th1 and Th2-associated immune response. Peptides inducing high IL-17 levels were also found in the present study. These results were consistent with those from other immunisation trials, resulting in the generation of cytokines involved in a mixed Th1/Th2 immune response [44], [48], [49]. IL-4 producing T-cell differentiation is an important step in developing an effective protective immune response. IL-4 can directly mediate worm expulsion mechanisms and is required for Th2 cell amplification and IL-5 also plays an import role in mediating host protection against helminth parasites [50]. The present results showed that some peptides such as B5, B6 and T14–17 plus AA0029, induced high IL-4 and IL-5 levels. It is worth noting that the protection induced by many vaccines against helminths, particularly *S. mansoni* experimental infection, has been associated with high IFN-γ and TNF-α production [51], [52]. TNF-α might play a role in accelerated worm expulsion through Th2 immune response enhancement [53] and IFN-γ production suppression may mediate parasite survival in fasciolosis [54]. The present study also found that the B1 peptide stimulated both TNF-α and IFN-γ, whilst peptides B5, T15 and T16 induced high IFN-γ levels, classically corresponding to a Th1-like immune response. This study strongly suggested that Th1 cytokines play a central role as biomarkers and should be used for measuring vaccination effectiveness. Some peptides evidently stimulated both IFN-γ and IL-4 cytokines, such as B5 and T15, suggesting their potential as vaccine candidates. Peptides B5 and T16 were the only ones having high IL-17 levels; however, the role of IL-17 has not yet been completely understood in *F. hepatica* infection. IL-17 has been associated with a severe form of the disease in experimental schistosomiasis models [55]. According to the data obtained in the present study, it can be hypothesised that immunological mechanisms participating in conferring protection against infection caused by *F. hepatica* involve Th1, Th2 and Th17 immune responses; the most protection-inducing peptides -according to survival rates, fluke burden and hepatic damage-, stimulated the production of high IFN-γ, IL-4 and IL-17 levels, respectively, after their administration with the AA0029 immunomodulator.

The specific role of CD4^+^ and CD8^+^ T-cells in protection against *F. hepatica* infection remains to be clearly elucidated. However, immunosuppression is one of the main mechanisms leading to liver fluke survival in a chronically-infected host, inducing a significant decrease in peripheral blood CD4^+^ and CD8^+^ T cells. Previous studies have demonstrated that *F. hepatica* cathepsin L has down-regulated CD4^+^ from the surface of human T-cells [56]. The more susceptible sheep host has shown reduced T-lymphocyte proliferation during *F. hepatica* infection [57]. Other authors have suggested lymphocyte migration, including CD4^+^ and CD8^+^ lymphocytes, from mesenteric lymph nodes to the antigen exposure site. CD8^+^ T-cells have been particularly evident in fibrotic areas of the liver during chronic infection [58]. It is well-known that helminth parasites are able to live for long periods in a host, immunoregulation being a key factor in this [59]. Some memory T-cells, including central memory T-cells and effector memory T-cells expressing CD197 (CCR7) and CD62L^+^, respectively, are involved in such immunoregulation [60]. However, it is not yet well understood how immunoregulation works in *F. hepatica*. Nevertheless, other studies have suggested the importance of central memory T-cells in establishing long-term immunity; central memory T-cells are maintained when the parasite is eliminated, but effector memory T-cells are not [61]. Vaccine effectiveness combines the action of both humoral and cellular immune responses; this is why central memory T-cells were also considered in the present study as a key target for peptide screening in the search for vaccine candidates. High CD62L^+^ expression levels have been shown to be protective in various infectious diseases [61], [62]. Importantly, higher CD62L^+^ percentages were obtained with T-epitopes in this study using either of the immunomodulators.

Most recent work aimed at developing an effective vaccine against *F. hepatica* has been based on recombinant antigens, parasite purified proteins, excretory/secretory released proteins and DNA-based vaccines. However, little is known concerning the development of subunit-based, chemically-synthesised anti-*F. hepatica* vaccines [63], [64]. This article has demonstrated that immunising CD1 mice with subunit-based and chemically-synthesised peptides containing B- or T-epitopes formulated in the ADAD vaccination system using the AA0029 immunomodulator led to obtaining protection against infection caused by *F. hepatica*, based on survival rates, reduced fluke burden and hepatic lesion score. Despite the use of a murine model for evaluating vaccine candidates against fasciolosis is still controversial, as mice are highly susceptible to the infection and animals could die during experimentation, the survival rate was here considered as a clear protection-inducing indicator, since all mice belonging to the unimmunised and infected control group, died between days 24 and 34 post-infection.

The present study’s findings have highlighted the immunoprophylactic potential of B- and T-cell epitope-containing synthetic peptides (B2, B5, B6 and T15) formulated in the ADAD vaccination system in a murine model. However, it should be noted that a deeper knowledge of the host-parasite interactions, and understanding the molecular and immunological mechanisms involved in inducing a protective response, might be crucial for better selecting the most appropriate vaccine formulation. Further studies aimed at studying the protection-inducing ability of the aforementioned peptides when formulated in combination, and tested in natural fasciolosis models, are in need.

## Supporting Information

Figure S1 *In vitro* cell viability using MTT assay after 48 h culture of mouse peritoneal macrophages cell line (J774.2) with B- and T-cell epitope-containing synthetic peptides. Synthetic peptides were assayed in a range from 1 to 100 µg/mL.
(TIF)

Figure S2 IgE antibody level detection in mice immunised with the synthetic peptides throughout the immunisation schedule. Data presented as box plots with the bottom and the top of the box indicating the 25^th^ and 75^th^ percentiles, respectively. A). Peptides formulated with the PAL immunomodulator. B). Peptides formulated with AA0029.
(TIF)

Figure S3 IgM antibody level detection in mice immunised with synthetic peptides throughout the immunisation schedule. Data presented as box plots with the bottom and the top of the box indicating the 25^th^ and 75^th^ percentiles, respectively. A). Peptides formulated with the PAL immunomodulator. B). Peptides formulated with AA0029.
(TIF)

Figure S4 Three-dimensional scatterplots represents cytokine levels induced by immunisation of mice with peptides containing B-cell epitopes. The Z-axis represents IFN-γ, IL-4, IL-10 and IL-17 levels for Figure A, B, C and D, respectively. The x and y axis represent CD197 and CD27 memory T-lymphocytes for each Figure. Blue indicates the use of AA0029 and green indicates PAL.
(TIF)

Figure S5 Three-dimensional scatterplots represents cytokine levels induced by immunisation of mice with peptides containing T-cell epitopes. The Z-axis represents IFN-γ, IL-4, IL-10 and IL-17 levels for Figure A, B, C and D, respectively. The x and y axis represent CD197 and CD27 memory T-lymphocytes for each Figure. Blue indicates the use of AA0029 and green indicates PAL.
(TIF)

Figure S6 Interaction plot for regulatory (A. IL-10), Th2 (B. IL-5, C. IL-4), Th17 (D. IL-17), Th1 (E. IL-2, F. IFN-γ) and innate inflammatory cytokine levels (G. IL-6, H. TNFα, I. IL-1α) elicited by epitope effect (B & T) and immunomodulator effect (AA0029 & PAL).
(TIF)
