# Supplementary figures and images for: In Vitro and In Vivo Studies for Assessing the Immune Response and Protection-Inducing Ability Conferred by Fasciola hepatica-Derived Synthetic Peptides Containing B- and T-Cell Epitopes
Source: PLoS One. 2014 Aug 14;9(8):e105323. doi: 10.1371/journal.pone.0105323 (PMC4133369; doi:10.1371/journal.pone.0105323)

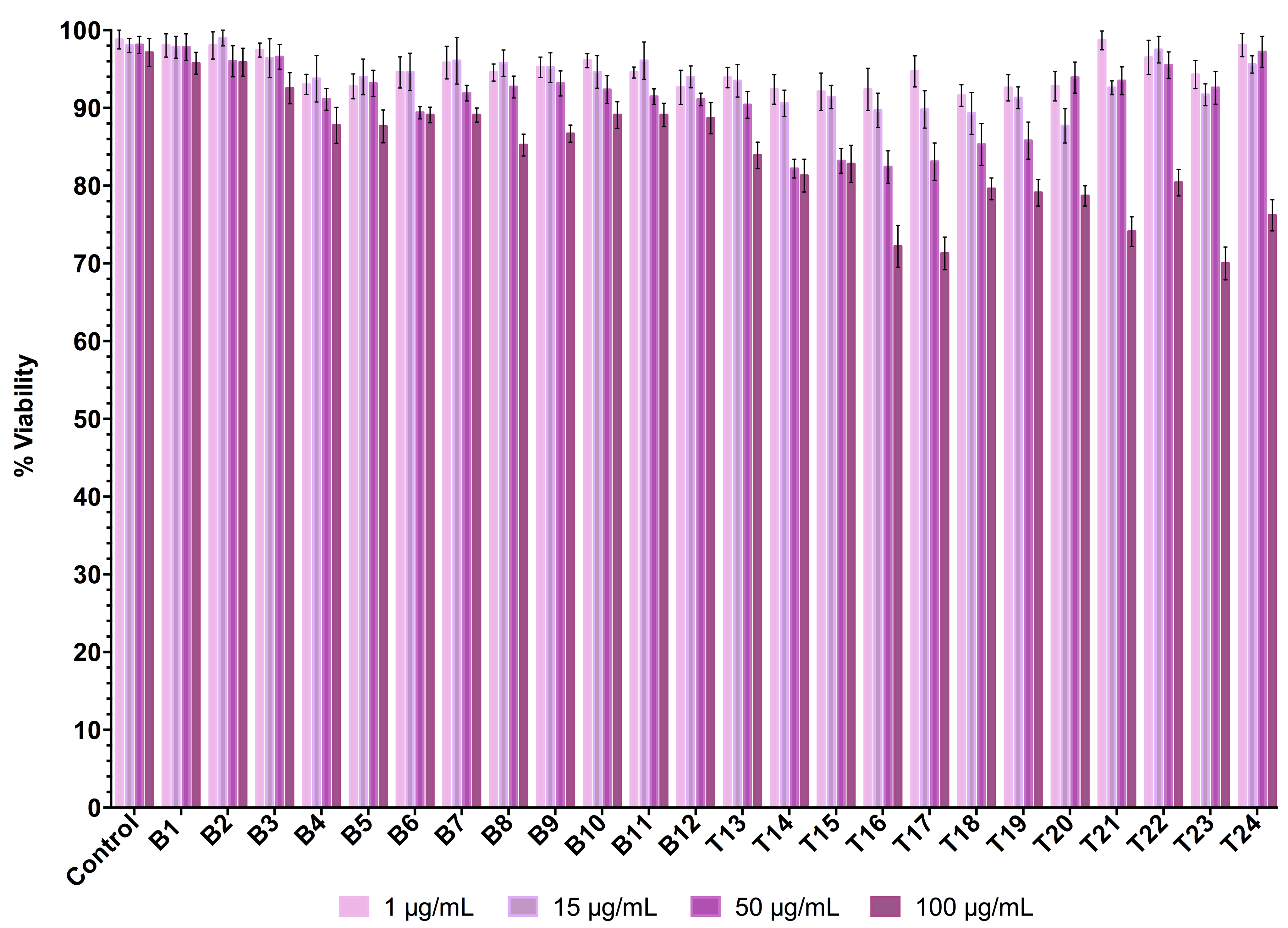

Supplement: Figure S1 — In vitro cell viability using MTT assay after 48 h culture of mouse peritoneal macrophages cell line (J774.2) with B- and T-cell epitope-containing synthetic peptides. Synthetic peptides were assayed in a range from 1 to 100 µg/mL. (TIF) [file pone.0105323.s001.tif]

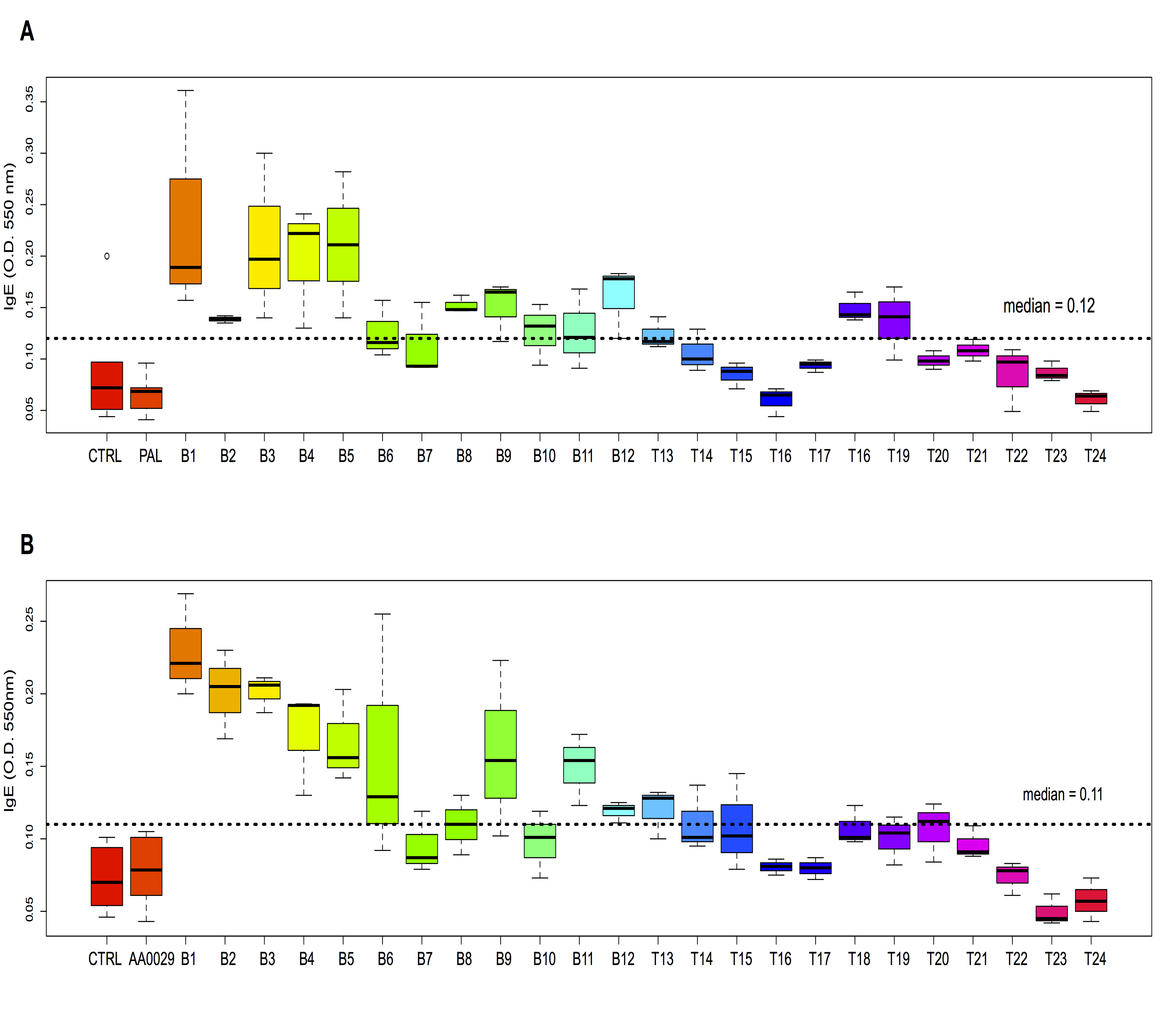

Supplement: Figure S2 — IgE antibody level detection in mice immunised with the synthetic peptides throughout the immunisation schedule. Data presented as box plots with the bottom and the top of the box indicating the 25th and 75th percentiles, respectively. A). Peptides formulated with the PAL immunomodulator. B). Peptides formulated with AA0029. (TIF) [file pone.0105323.s002.tif]

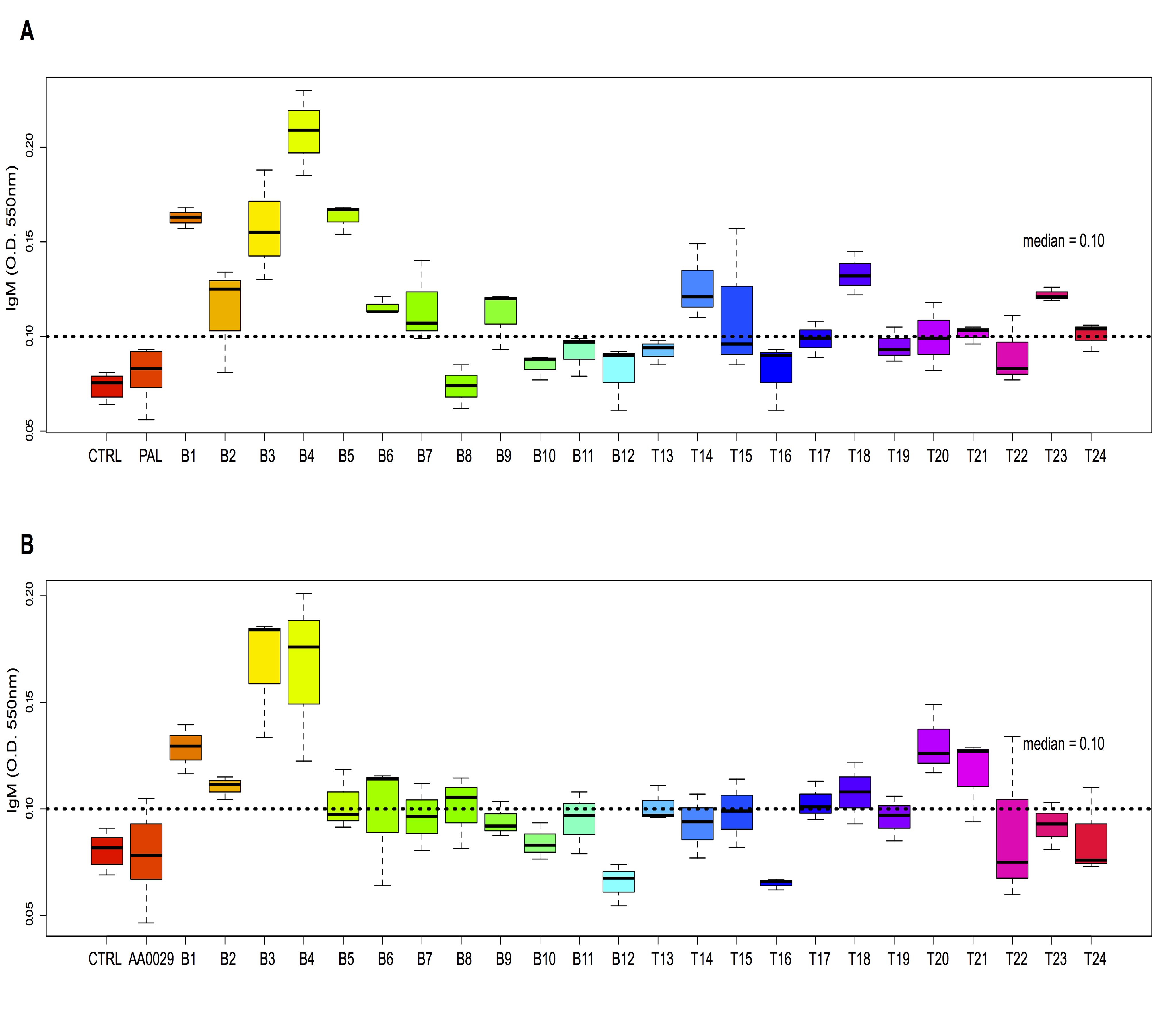

Supplement: Figure S3 — IgM antibody level detection in mice immunised with synthetic peptides throughout the immunisation schedule. Data presented as box plots with the bottom and the top of the box indicating the 25th and 75th percentiles, respectively. A). Peptides formulated with the PAL immunomodulator. B). Peptides formulated with AA0029. (TIF) [file pone.0105323.s003.tif]

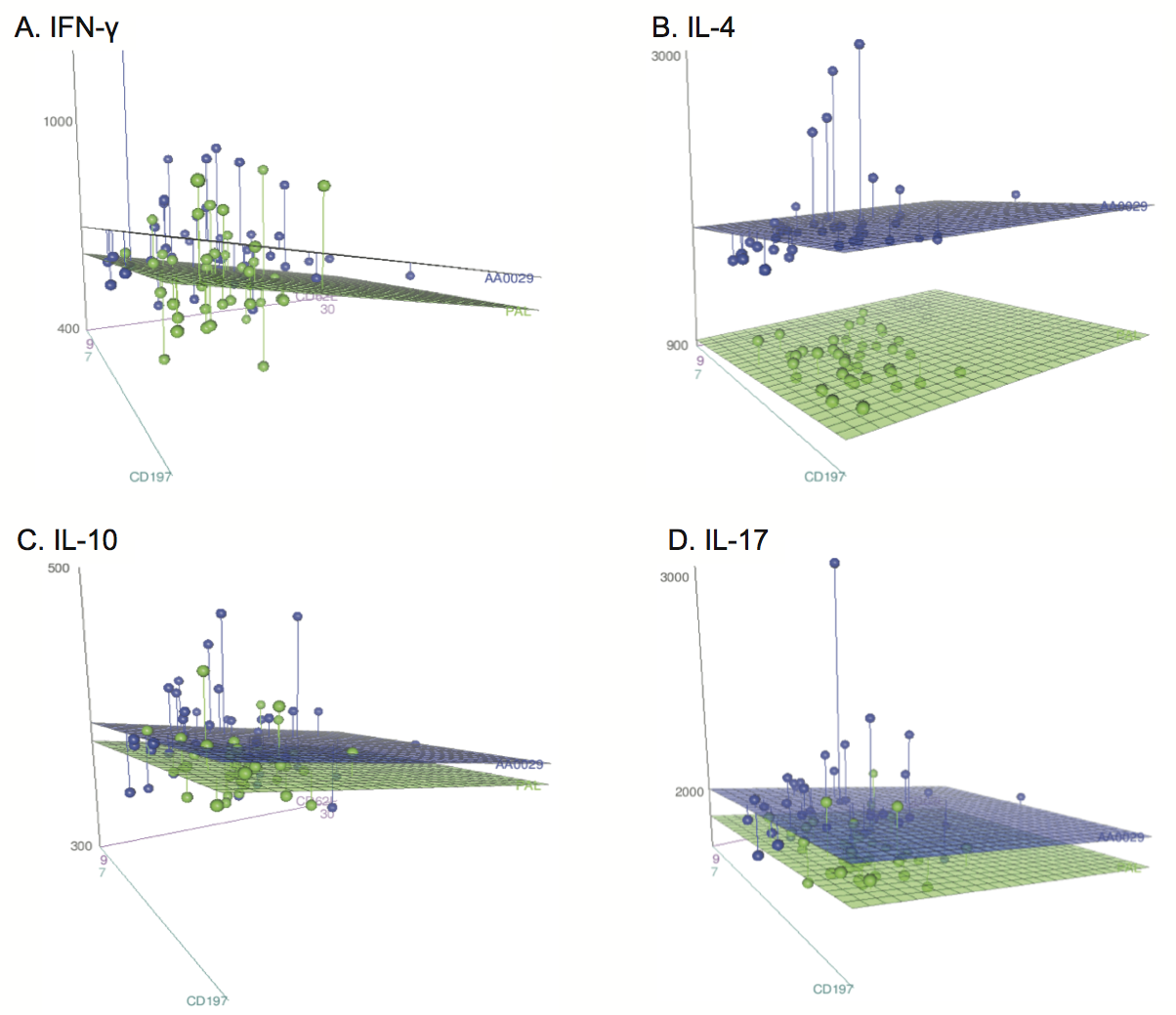

Supplement: Figure S4 — Three-dimensional scatterplots represents cytokine levels induced by immunisation of mice with peptides containing B-cell epitopes. The Z-axis represents IFN-γ, IL-4, IL-10 and IL-17 levels for Figure A, B, C and D, respectively. The x and y axis represent CD197 and CD27 memory T-lymphocytes for each Figure. Blue indicates the use of AA0029 and green indicates PAL. (TIF) [file pone.0105323.s004.tif]

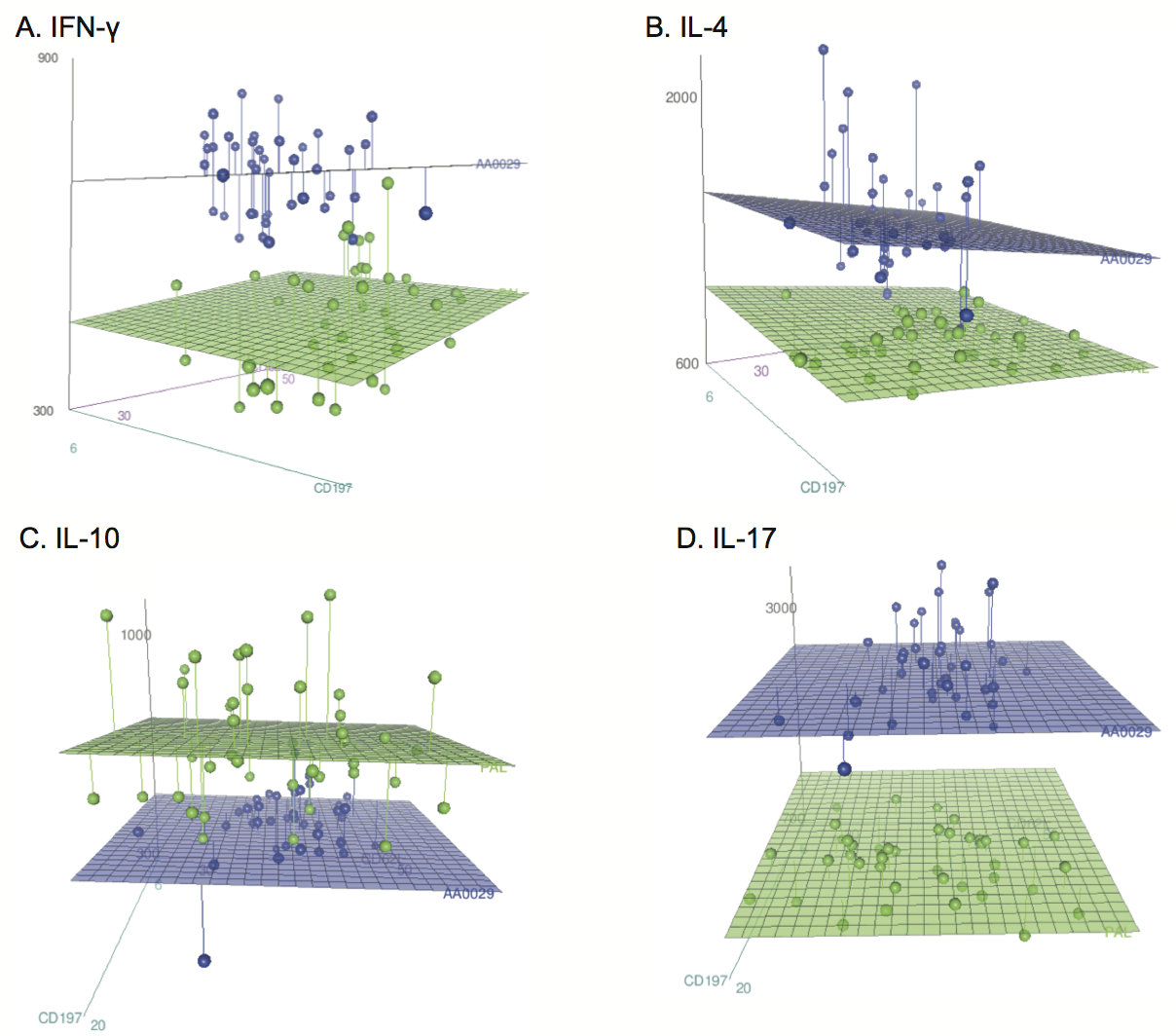

Supplement: Figure S5 — Three-dimensional scatterplots represents cytokine levels induced by immunisation of mice with peptides containing T-cell epitopes. The Z-axis represents IFN-γ, IL-4, IL-10 and IL-17 levels for Figure A, B, C and D, respectively. The x and y axis represent CD197 and CD27 memory T-lymphocytes for each Figure. Blue indicates the use of AA0029 and green indicates PAL. (TIF) [file pone.0105323.s005.tif]

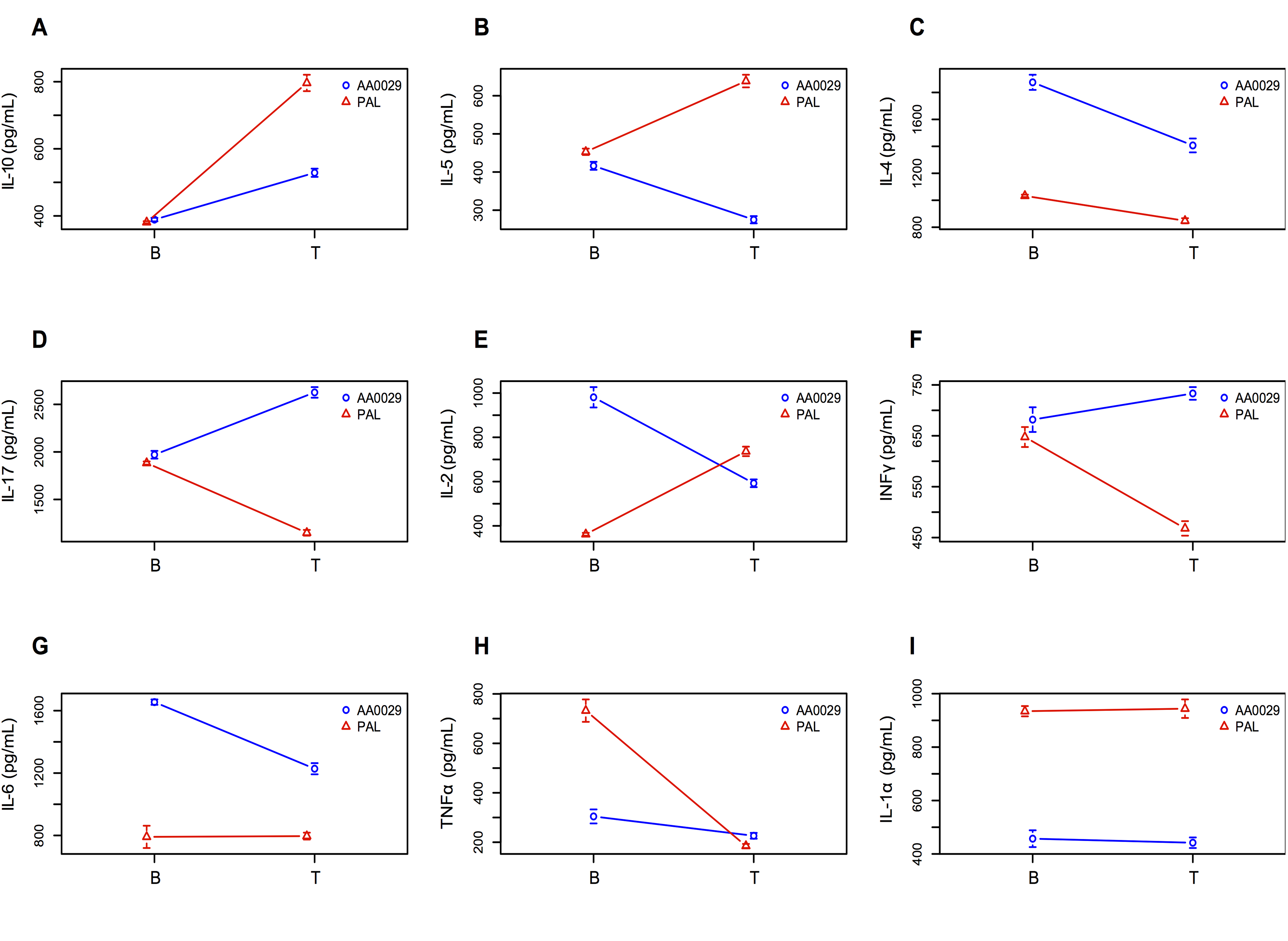

Supplement: Figure S6 — Interaction plot for regulatory (A. IL-10), Th2 (B. IL-5, C. IL-4), Th17 (D. IL-17), Th1 (E. IL-2, F. IFN-γ) and innate inflammatory cytokine levels (G. IL-6, H. TNFα, I. IL-1α) elicited by epitope effect (B & T) and immunomodulator effect (AA0029 & PAL). (TIF) [file pone.0105323.s006.tif]
